# Identification and Characterization of the Bioactive Polyphenols and Volatile Compounds in Sea Buckthorn Leaves Tea Together With Antioxidant and α-Glucosidase Inhibitory Activities

**DOI:** 10.3389/fnut.2022.890486

**Published:** 2022-04-29

**Authors:** Ningning Wang, Xiufeng Wen, Yan Gao, Shunguang Lu, Yimeng Li, Yanbin Shi, Zhigang Yang

**Affiliations:** ^1^School of Pharmacy, Lanzhou University, Lanzhou, China; ^2^Seabuckthorn Development and Management Center of the Ministry of Water Resources, Beijing, China; ^3^Collaborative Innovation Center for Northwestern Chinese Medicine, Lanzhou University, Lanzhou, China

**Keywords:** polyphenols, volatile compounds, sea buckthorn leaves tea, LC-MS, HS-SPME-GC-MS, antioxidant activity, α-glucosidase inhibitory activity

## Abstract

Sea buckthorn leaves have been used for tea making in food field gradually. This study was carried out to characterize the bioactive polyphenols and volatile compounds in sea buckthorn leaves (SL), sea buckthorn leaves green tea (SGT), and sea buckthorn leaves black tea (SBT) by using high-performance liquid chromatography with an UV detector (HPLC-UV), the liquid chromatography-mass spectrometry (LC-MS), and headspace solid-phase microextraction coupled with gas chromatography-mass spectrometry (HS-SPME-GC-MS), in combination with multivariate analysis. A total of 48 non-volatile metabolites and 295 volatile metabolites were identified. Then, the total polyphenol and total flavonoid contents in SL, SGT, and SBT were also analyzed. Moreover, SL and SGT showed greater antioxidant activities based on 2,2-diphenyl-1-picrylhydrazyl (DPPH), 2,2'-azino-bis(3-ethylbenzothiazoline-6-sulfonic acid) diammonium salt (ABTS), and oxygen radical absorbance capacity (ORAC) results. At the concentration of 0.1 mg/ml, their DPPH and ABTS radical scavenging ratios were 66 to 95%, while SBT exhibited lower antioxidant activity of 26 to 44%. SL, SGT, and SBT displayed moderate α-glucosidase inhibitory activity.

## Introduction

*Hippophae rhamnoides* L., commonly called as sea buckthorn, is a hardy deciduous shrub or tree widely distributed in northwest, northeast, and southwest of China. Its fruits have a variety of effects, including relieving cough, eliminating phlegm, strengthening spleen, eliminating food, promoting blood circulation, and disperses stasis (1). Extensive studies have shown that the leaves and fruits contain rich of bioactive substances, such as flavonoids, phenolic compounds, and ascorbic acid, which are expected to exert strong antioxidant effects (2). Furthermore, the total contents of polyphenol and flavonoids in sea buckthorn leaves are higher than that in the fruits (3). However, the sea buckthorn leaves resources have not being used properly. China has the richest natural sea buckthorn germplasm resources in the world, as well as the largest area under artificial cultivation, with a total planting area of 2.15 million hm^2^. Several studies have shown that the sea buckthorn leaf is a non-toxic substance, the LD50 of its extract was more than 10 g/kg body weight of rats (4). Simultaneously, sea buckthorn leaves have been listed as a new resource for food management in 2013 in China. Sea buckthorn leaves have more advantages in developing functional food, owing to their high content of active ingredients. It has been developed into tea products in food field gradually. It is of great significance to determine their phytochemicals and bioactivities for promoting the full development and utilization of sea buckthorn leaves resources.

Processing methods are considered to be the main factors affecting the biotransformation of phytochemicals in tea, leading to the diversity of tea flavor and bioactivity (4). However, the non-volatile components and volatile organic components (VOCs) are not clearly in sea buckthorn leaves tea from different processing. Liquid chromatography-mass spectrometry (LC-MS) has been widely used in the phytochemical study, due to its high resolution and sensitivity (5). As one of the most commonly used methods for fingerprint analysis, high-performance liquid chromatography (HPLC) has the advantages of fast analysis, high separation efficiency, and wide application (6). Aroma is one of the important factors in the evaluation of tea quality. The headspace solid-phase microextraction (HS-SPME) has obvious advantage of simple operation, high sensitivity, and no secondary pollution. HS-SPME combined with gas chromatography-mass spectrometry (GC-MS) is one of the most widely used detection technologies in tea aroma compounds analysis (7). The multivariate statistical analysis can simplify the dimension of high-dimensional and complex data on the basis of retaining the original information to the greatest extent and establish a reliable mathematical model to summarize the characteristics of the metabolic spectrum of the study object. The multivariate statistical analyses, such as principal component analysis (PCA), hierarchical cluster analysis (HCA), and orthogonal partial least squares discriminant analysis (OPLS-DA), have been performed to determine the different metabolites (8).

In this study, to verify the differences of non-volatile and volatile metabolites in SL, SGT, and SBT, twelve batches of sea buckthorn leaves tea were analyzed by HPLC, LC-MS, and HS-SPME-GC-MS. To the best of our knowledge, the non-volatile and volatile metabolites profile, together with their antioxidant and α-glucosidase inhibitory activities of sea buckthorn leaves tea were first reported.

## Materials and Methods

### Chemicals and Materials

Isoquercetin (>98%) and narcissoside (>98%) were purchased from Chengdu MadDesheng Technology Corporation Ltd. Kaempferol-3-O-rutinoside (>98%) and quercitrin (>98%) were purchased from Chengdu Pufield Biotechnology Corporation Ltd. Epigallocatechin (>98%), epicatechin (>98%), quercetin (>98%,), isorhamnetin (>98%), epigallocatechin gallate (EGCG) (>98%), (-)-epicatechin gallate (ECG) (>98%), and corosolic acid (>98%) were purchased from Shanghai Shidande Biotechnology Corporation Ltd. Methanol and acetonitrile were LC/MS grade and purchased from Merck Company (Germany). 2,2-diphenyl-1-picrylhydrazyl (DPPH), 2,2'-azino-bis(3-ethylbenzothiazoline-6-sulfonic acid) diammonium salt (ABTS), α-glucosidase, 3,4,5-trihydroxybenzoic acid (gallic acid), butylated hydroxyl toluene (BHT), ascorbic acid (vitamin C), 2,2'-azobis(2-methylpropionamidine) dihydrochloride (AAPH), 6-hydroxy-2,5,7,8-tetramethylchroman-2-carboxylic acid (Trolox), fluorescein, and phosphate-buffered saline (PBS) were purchased from Solaibao Technology Corporation Ltd.

**Figure 1:**
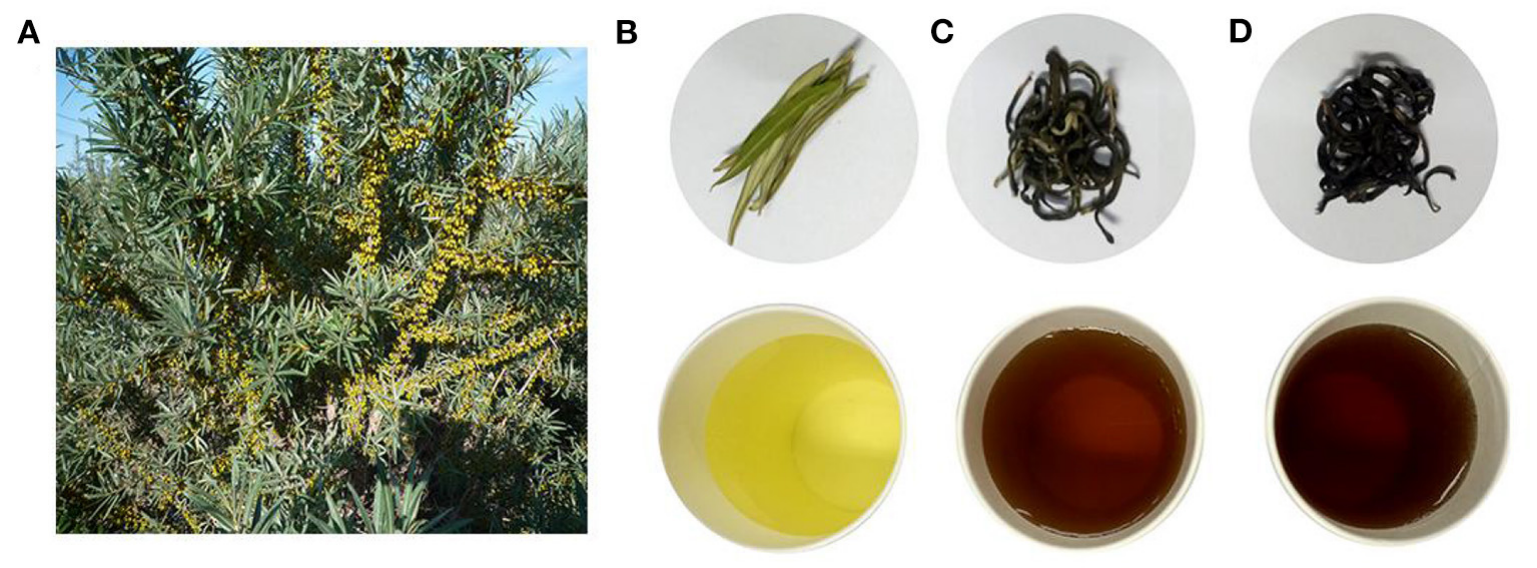
Pictures of sea buckthorn leaves tea. **(A)** *Hippophae rhamnoides* L. (sea buckthorn). **(B)** Sea buckthorn leaves (SL). **(C)** Sea buckthorn leaves green tea (SGT). **(D)** Sea buckthorn leaves black tea (SBT) (top, dry leaves; bottom, tea infusion).

Twelve tea samples (Supplementary Table S1), including SL, SGT, and SBT, were purchased from a company in Xinjiang, China. The processing technology of sea buckthorn leaves tea as following: the fresh sea buckthorn leaves were naturally dried at room temperature after picking. SGT were made from fresh sea buckthorn leaves by rolling, screening, drying, while fresh sea buckthorn leaves were made into SBT by withering, rolling, fermentation, and drying process. All the samples were identified as sea buckthorn leaves tea (No.202109001-No.202109012) by Zhigang Yang, which were stored in the School of Pharmacy Lanzhou University. The images of sea buckthorn leaves tea samples are shown in Figure 1.

### Sample Preparation

All the dried sea buckthorn tea samples were crushed milled into sieve (50 meshes). 0.25 g of tea power was weighed and placed into a plug colorimetric tube. Then, 25 ml of 70% methanol was added for ultrasonic extraction for 30 min at 30°C using a ultrasonic cleaner (KQ5200E, Kunshan Ultrasonic Instruments Corporation Ltd., China). The extract was centrifuged at 13,000 rpm/min for 2 min. Afterward, the supernatants filtered through a 0.22-μm nylon filter for LC-MS and HPLC analysis. The filtrate was diluted for 100 times with methanol to prepare sample solution for total polyphenol content (TPC), total flavonoid content (TFC), antioxidant, and α-glucosidase inhibitory activities.

### Liquid Chromatography-Mass Spectrometry Analysis

The LC-MS analysis of tea samples was based on the previous methods (9). The column was CORTECS UHPLC C18 Column (2.1 mm × 100 mm, 1.6 μm), Waters, USA. The mobile phase consisted of 0.1% aqueous formic acid (A) and 0.1% acetonitrile (B); the gradient elution was as follows: 0–2 min, 7% B; 2–3 min, 7–12% B; 3–8 min, 12–18% B; 8–12 min, 18–60% B; 12–13 min, 60–100% B; and 13–14 min, 100% B. Samples (2 μl) were eluted at 0.3 ml/min and the column oven was kept at 36°C. Mass spectrometry conditions were as follows: electrospray ion source (ESI), negative ion detection mode acquisition, and scanning range of m/z 100–1,700. ESI source parameters were as follows: dry gas (N_2_) flow rate of 5 ml/min, dry gas temperature of 300°C, atomization gas pressure of 35 psig, capillary voltage of 3,500 V, nozzle voltage of 1,000 V, capillary outlet voltage of 400 V, and collision energy of 20 eV. The data processing software was the Agilent Profinder software (B.08.00, Agilent, USA) and Mass Profiler Professional (MPP) software (version 14.9, Agilent); processing included noise filtering, molecular feature extraction (MFE), peak alignment, and normalization.

### High-Performance Liquid Chromatography Fingerprint Analysis

The HPLC system consisted of EasySep-3030 liquid chromatography, EasySep-3030 UV detector, AS-2000 automatic sampler, EasySep-3030 binary pump, and C18 column (4.6 mm × 250 mm, 5 μm), which were purchased from Shanghai Tong Micro Company. The mobile phase consisted of acetonitrile (A) and 0.4% (v/v) phosphate water (B). The gradient elution was as follows: 0–40 min, 10–20% A; 40–50 min, 20–40% A; and 50–60 min, 40–80% A. Samples (10 μl) were eluted at 0.8 ml/min and the column temperature was maintained at 30°C. The detection wavelength was 280 nm.

High-performance liquid chromatography chromatographic data of 12 batches of tea samples were imported in cdf format into the “TCM Chromatography Fingerprint Similarity Software Evaluation System (2012 version)” software developed by the National Pharmacopeia Commission, the average method was set, and the time window width was 0.2 min. After multipoint correction and peak matching, the control fingerprinting profiles of SL, SGT, and SBT were generated, respectively.

### Data Processing

The SIMCA 14.1 software (Umetrics, Umea, Sweden) was used for a series of multivariate data analysis. Principal component analysis (PCA) is an unsupervised pattern recognition that can observe the data set and display the similarities and differences. Orthogonal partial least squares discriminant analysis (OPLS-DA) is used to analyze the metabolic pattern, which is a supervised pattern recognition used for analyzing the intrinsic variation of the data. R^2^Y and Q^2^Y of the model represent model interpretability of variable Y and predictability, respectively. The OPLS-DA model was validated by permutation tests (*n* = 200).

### Headspace Solid-Phase Microextraction Coupled With Gas Chromatography-Mass Spectrometry Analysis

1 g of the tea powder was transferred to a 20-Ml Headspace Vial (Agilent, Palo Alto, California, USA), Containing NaCl Saturated Solution. The Vials Were Sealed Using Crimp-Top Caps With Tetrafluoroethylene (TFE)-Silicone Headspace Septa (Agilent). At the Time of SPME Analysis, Each Vial Was Placed in 100°C for 5 min and Then a 120 μm Divinylbenzene/Carboxen/Polydimethylsiloxane Fiber (Agilent) Was Exposed to the Headspace of the Sample for 15 min at 100°C.

After sampling, desorption of the volatile organic compounds (VOCs) headspace from the fiber coating was carried out in the injection port of the GC apparatus (Model 8890, Agilent) at 250°C for 5 min in the splitless mode. The identification and quantification of VOCs were carried out using an Agilent Model 8890 GC and a 5977B mass spectrometer (Agilent), equipped with a DB-5MS (5% phenyl-polymethylsiloxane) capillary column (30 m × 0.25 mm, 0.25 μm). Helium was used as the carrier gas at a linear velocity of 1.2 ml/min. The injector temperature was kept at 250°C and the detector temperature was kept at 280°C. The oven temperature was programmed from 40°C (3.5 min), increasing at 10°C/min to 100°C, at 7°C/min to 180°C, at 25°C/min to 280°C, hold for 5 min. Mass spectra were recorded in electron impact (EI) ionization mode at 70 eV. The quadrupole mass detector, ion source, and transfer line temperatures were set, respectively, at 150, 230, and 280°C. Mass spectra were scanned in the range m/z 50–500 at 1 s intervals.

The volatile compounds were identified by comparing the mass spectra with the data system library [Metware gas chromatography (MWGC) or National institute of standards and technology (NIST)] and linear retention index. The data were unit variance scaled before unsupervised PCA. Significantly regulated metabolites between the groups were determined by Variable importance in the projection (VIP) ≥ 1 and absolute log2FC (fold change) ≥ 1. VIP values were extracted from OPLS-DA result, which also contain score plots and permutation plots, generated using R package MetaboAnalystR. The data was log transform (log 2) and mean centering before OPLS-DA. In order to avoid overfitting, a permutation test (200 permutations) was performed.

### Determination of Total Polyphenol Content

Total polyphenol content (TPC) was quantified using the Folin–Ciocalteu colorimetric assay with gallic acid as the standard (10). Briefly, 100 μl sample solution was mixed with 50 μl Folin-Ciocalteu's phenol solution (0.5 N). After 5 min, 100 μl Na_2_CO_3_ (75 mg/ml) solution was added to avoid light reaction. The absorbance was measured at 765 nm using a microplate reader after 60 min.

### Determination of Total Flavonoid Content

Total flavonoid content (TFC) was determined using the colorimetric method with rutin as the standard (11). Briefly, 20 μl sample solution and 20 μl Na_2_NO_2_ (30 mg/ml) were mixed and incubated at room temperature for 6 min. Then, 20 μl Al(NO_3_)_3_ solution (60 mg/ml) was added and reacted for 6 min. Afterward, 140 μl NaOH solution was added and mixed well. The absorbance was measured at 510 nm using a microplate reader after 15 min.

### Antioxidant Activity

The antioxidant activities were investigated by DPPH, ABTS, and oxygen radical absorbance capacity (ORAC) assays according the method described with some modifications (12). Briefly, 100 μl sample solution was taken to a 96-well plate and 100 μl DPPH solution was added to mix thoroughly. The absorbance was measured at 517 nm after 30 min. The 50 μl sample solution was put on a 96-well plate and 200 μl ABTS solution was added to mix thoroughly. The absorbance value was measured at 734 nm after 6 min. VC and BHT were used as positive controls. 20 μl sample solution and 120 μl fluorescein were transferred to a 96-well black microplate and then incubated for 15 min at 37°C. After 60 μl AAPH solution was added, fluorescence was immediately read with an excitation wavelength of 485 nm and an emission at 538 nm. Trolox was used as the control.

### α-Glucosidase Inhibitory Activity

The α-glucosidase inhibition activities were carried out using the method described for some modifications (12). Firstly, 100 μl sample and 50 μl α-glucosidase (1.3 U/ml) were added to the 96-well plate. Then, 50 μl p-nitrophenyl-beta-D-glucuronide (PNPG) (2.5 mmol/l) was added for 30 min at 37°C and 80 μl Na_2_CO_3_ (0.2 mol/l) was added to stop the reaction. The absorbance value was determined at 405 nm. Acarbose was used as positive control.

### Statistical Analysis

Total polyphenol content, total flavonoid content, antioxidant activity, and α-glucosidase inhibitory activity of SL, SGT, and SBT were separated using the Tukey's honestly significant difference (HSD) test after one-way ANOVA. Data were presented as mean ± SD, calculated from three replicates. All the statistical analyses were carried out using SPSS software version 22.0.

## Results and Discussions

### Results of the Liquid Chromatography-Mass Spectrometry Analysis

**Figure 2:**
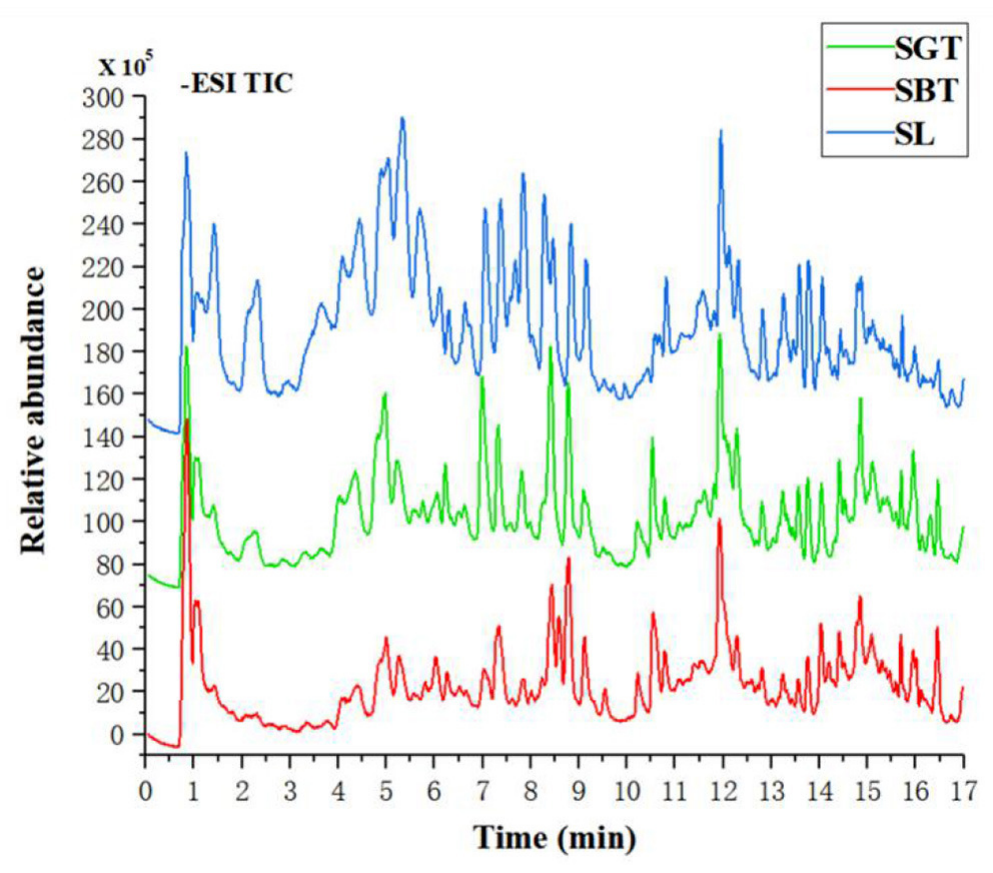
The total ion chromatograms of SL, SGT, and SBT of liquid chromatography-mass spectrometry (LC-MS) in the negative ion mode.

Non-Volatile metabolites in sea buckthorn leaves tea samples were identified according to authentic standards and comparisons with the literature (13, 14). A total of 48 compounds were identified in SL, SGT, and SBT, including 11 tannins, 27 flavonoids, and 4 phenolic acids (Table 1). Tea polyphenols, the main active components in tea, such as gallocatechin, epigallocatechin, catechin, epicatechin, epigallocatechin gallate, and (-)-epicatechin gallate, were also detected in sea buckthorn leaves tea. Recent studies have well documented that polyphenols have the benefits for human health with antioxidant, anti-inflammation, anticancer, anticardiovascular, antimicrobial, antihyperglycemic, and antiobesity properties (15). The sea buckthorn leaves tea is mainly composed of flavonoid glycosides with isorhamnetin, quercetin, and kaempferol as the aglycone and hydrolyzed tannins. Previous studies also have shown that sea buckthorn leaves contain a large amount of flavonol glycosides of isorhamnetin and quercetin derivatives and ellagic tannins (16, 17). In the total ion current chromatograms, most of the peak of SBT rapid decreased, such as gallocatechin, catechin, stachyurin, quercetin dihexoside, hippophaenin B, and epicatechin dramatically declined (Figure 2 and Table 1). The content of flavonoid glycosides in sea buckthorn leaves decreased during black tea processing, suggesting that the processing technology of green tea had little effect on the chemical constituents of sea buckthorn leaves and the fermentation technology needed to be further optimized. The sensation of bitterness and astringency are important sensory characteristics of tea. The flavonoid glycosides are rich in sea buckthorn leaves, especially isorhamnoside, which are closely related to bitterness and astringency (18). After fermentation processing, sea buckthorn leaves may significantly decrease the content of flavonoid glycosides to further reduce astringency and improved taste. Hence, reduction of the flavonol glycosides in SBT had a lower sensory threshold for astringency that may suitable for development into black tea drink.

**Table 1:** Information of main nonvolatile compounds from sea buckthorn leaves tea.

**No**.	**RT(min)**	**[M-H]^**−**^(m/z)**	**Formula**	**MS/MS ions**	**Tentative identification**
1	0.843	191.0569	C_7_H_12_O_6_	173,155,111	Quinic acid
2	1.024	331.0681	C_13_H_16_O_10_	271,169,125	Gallic acid hexoside
3	1.128	169.0149	C_7_ H_6_ O_5_	125	Gallic acid
4	1.462	305.0673	C_15_H_14_O_7_	287,261,219,179,137	Gallocatechin
5	2.323	783.0718	C_34_H_24_O_22_	301,481	Pedunculagin
6	2.654	305.0673	C_15_H_14_O_7_	287,261,219,179,137	Epigallocatechin ^*^
7	3.039	577.1366	C_30_H_26_ O_12_	425,407,289,125	Procyanidin B2
8	3.341	577.1366	C_30_H_26_O_12_	425,407,289,125	B-type procyanidin dimer
9	3.449	183.0304	C_8_ H_8_O_5_	124	Methyl gallate
10	3.532	289.0725	C_15_H_14_O_6_	245,205,179	Catechin
11	4.309	633.0757	C_27_H_22_O_18_	463,301,275,169	Gallic-hexahydroxybibenzoyl-hexose
12	4.311	935.0840	C_41_H_28_O_26_	633,275	Stachyurin
13	5.123	625.1432	C_27_H_30_O_17_	463,301	Quercetin-dihexoside
14	5.321	1103.0907	C_48_H_32_O_31_	1059,935,787,633	Hippophaenin B
15	5.556	785.0870	C_34_H_26_O_22_	633,483,300	Tellimagrandin I
16	5.802	289.0725	C_15_H_14_O_6_	271,245,205,179,165	Epicatechin^*^
17	6.001	457.0790	C_22_H_18_O_11_	331,305,169	Epigallocatechin gallate^*^
18	6.084	635.0914	C_27_H_24_O_18_	483,465,313	1,2,6-Tri-O-galloyl-β-d-glucopyranose
19	6.249	755.2069	C_33_H_40_O_20_	609,431,285,227	Kaempferol-3-O sophorose-7-O-rhamnoside
20	6.280	639.1589	C_28_H_32_O_17_	477,315	Isorhamnetin-3,7-O-hexglycoside
21	6.627	785.2176	C_34_H_42_O_21_	639,460,315	Isorhamnetin-3-O-sophora-7-O-rhamnoside
22	6.945	935.0840	C_41_H_28_O_26_	633,463,300	Casuarictin
23	7.293	609.1485	C_27_H_30_O_16_	463,447,301,271	Quercetin-hexose-rhamnose
24	7.442	755.2069	C_33_H_40_O_20_	609,463,446,301,300	Quercetin-3-O-glucose rhamnose-7-O-rhamnoside
25	7.827	433.0430	C_20_H_18_O_11_	301	Quercetin-3-O-arabinoside
26	8.199	1085.0807	C_48_H_30_O_30_	633,450,425,299	Ellagotannin
27	8.221	977.2617	C_44_H_49_O_25_	831,771,625,447,301	Quercetin-sinapoyl-sophoroside-rhamnoside
28	8.320	300.9985	C_14_H_6_O_8_	283,257,229,185,157	Ellagic acid
29	8.370	739.2119	C_33_H_40_O_19_	593,447,285	Kaempferol-3-O-rutin-7-O-rhamnoside
30	8.446	593.1533	C_27_ H_30_ O_15_	431,285	Lonicerin
31	8.463	593.1533	C_27_H_30_O_15_	447,285,227	Kaempferol-3-O-glucose rhamnoside
32	8.499	623.1645	C_28_H_32_O_16_	477,461,315	Isorhamnetin-3-O-glucose-7-O-rhamnoside
33	8.668	441.0843	C_22_H_18_O_10_	289,169	(-)-Epicatechin gallate^*^
34	8.701	609.1488	C_27_H_30_O_16_	301,151	Quercetin-3-O-rutinoside
35	8.767	623.1629	C_28_H_32_O_16_	477,461,315,300,151	Isorhamnetin-3-O-rutinoside
36	9.049	463.0901	C_21_H_20_O_12_	301,271	Isoquercitrin^*^
37	9.082	593.1534	C_27_H_30_O_15_	461,447,315,300	Isorhamnetin-pentose-rhamnoside
38	9.082	463.0901	C_21_H_20_O_12_	300,271	Hyperoside
39	9.140	991.2764	C_42_H_55_O_27_	845,639,315	Isorhamnetin-3-O-sophorose-O-acetyl glucose−7-O-rhamnoside
40	9.296	961.2666	C_40_H_50_O_27_	815,639,461,315	Isorhamnetin-3-O-sophorose-O-glucuronic acid-7-O-rhamnoside
41	10.159	593.1533	C_28_H_32_O_16_	285	Kaempferol-3-O-rutinoside^*^
42	10.490	447.095	C_21_H_20_O_11_	301,271,179,151	Quercitrin ^*^
43	10.490	623.164	C_28_H_32_O_16_	315,300,271,151	Isorhamnetin-3-O-rutinoside
44	10.509	623.1638	C_28_H_32_O_16_	315	Narcissus glycosides^*^
45	10.571	447.0950	C_21_ H_20_O_11_	301,284,151	Astragalin
46	10.761	477.1062	C_22_H_22_O_12_	315,300,271,151	Isorhamnetin-3-O-glucoside
47	11.377	447.0950	C_21_ H_20_O_11_	301,151	Quercetin-7-rhamnoside
48	15.098	471.3494	C_30_H_48_O_4_	393	Cosolic acid^*^

* *The compounds with asterisk were identified by authentic standards*.

### Results of the High-Performance Liquid Chromatography Fingerprint Analysis

Comprehensive validation of the present method, including precision, repeatability, and stability, relative standard deviation (RSD) of relative retention time (RRT) was 0.48, 0.96, and 0.86%, respectively; RSD of relative peak area (RPA) was 0.96, 4.68, and 0.40%, respectively. These results showed that the established approach was suitable for analysis. After multipoint correction and peak matching, the control fingerprint profiles of SL, SGT, and SBT were generated and 14 common peaks were generated. The similarity between SL and control fingerprint was at 0.972–0.986. The similarity between SGT and control fingerprints was at 0.886–0.980. The similarity between SBT and control fingerprints was at 0.404–0.455. These results showed that the composition of SL and SGT was not obvious changed after frying process, while the chemical composition of SBT varied greatly after fermentation process of SL.

**Figure 3:**
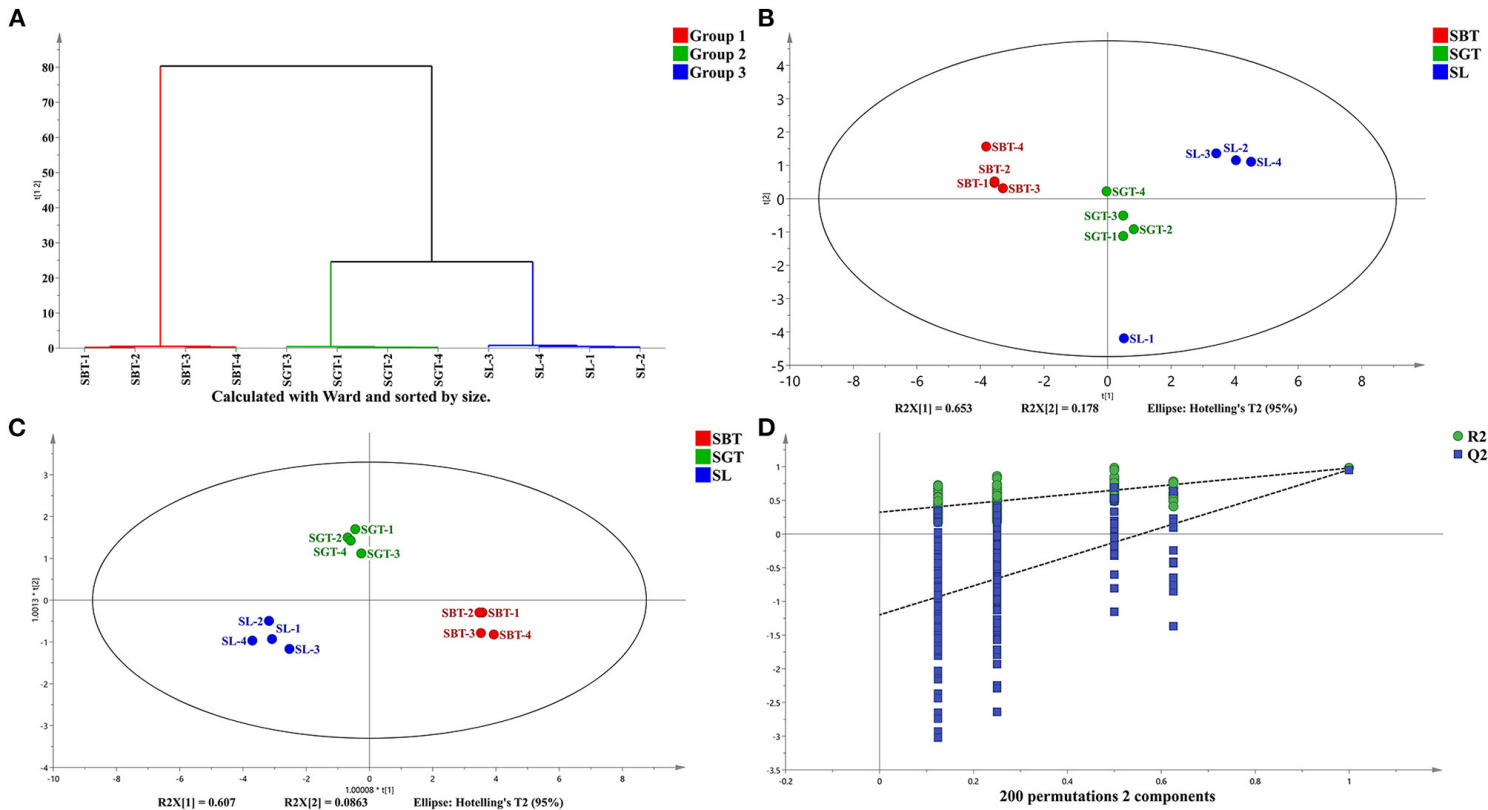
High-performance liquid chromatography (HPLC) analysis of SL, SGT, and SBT. **(A)** Cluster analysis diagram. **(B)** Principal component analysis (PCA) score plot. **(C)** Orthogonal partial least squares discriminant analysis (OPLS-DA) score plot. **(D)** Permutation plot of OPLS-DA (R^2^X = 0.969; R^2^Y = 0.97; and Q^2^ = 0.932).

Cluster analysis and common peak relative peak area data were imported into the SIMCA 14.1 software to cluster analysis of sea buckthorn tea samples and the classification distance between samples was calculated using the Ward method. As shown in Figure 3A, the SL, SGT, and SBT samples were clustered into three independent classes and the distance between the SL and SBT was far away, which showed that there were certain differences among the sea buckthorn leaves tea samples under different processing methods.

Principal component analysis (PCA), cluster analysis, and OPLS-DA were used to classify the volatile components of SL, SGT, and SBT and similar samples were gathered together to find the relevant material by analyzing the score and load value. The cumulative contribution rate of the first two principal components is 83.1%, which can reflect the information of most of the characteristic peaks (Figure 3B). The nonvolatile of SL, SGT, and SBT was significantly separated (Figures 3B,C), indicating that the chemical composition content varied greatly between the samples from different processing and the results were consistent with the similarity analysis and cluster. The OPLS-DA model was validated by the two hundred random permutation tests (Figure 3D) and no overfitting was observed according to the results. Consequently, the model could be used to distinguish the sea buckthorn leaves tea among different processing of SL, SGT, and SBT. The HPLC characteristic fingerprint will benefit the authentication and quality evaluation of sea buckthorn leaves tea samples.

### Results of the Headspace Solid-Phase Microextraction Coupled With Gas Chromatography-Mass Spectrometry Analysis

QC samples are used to detect the state of the instrument before sampling and to evaluate the stability of the system throughout the experiment. The peak retention time and peak area of QC samples in total ion chromatograms (TICs) overlapped indicating that the instrument was stable (**Figure 5A**). The PCA analysis of all the samples showed that QC had the distribution within ± 2 SD, which also suggested the stability and high quality of the data.

**Figure 4:**
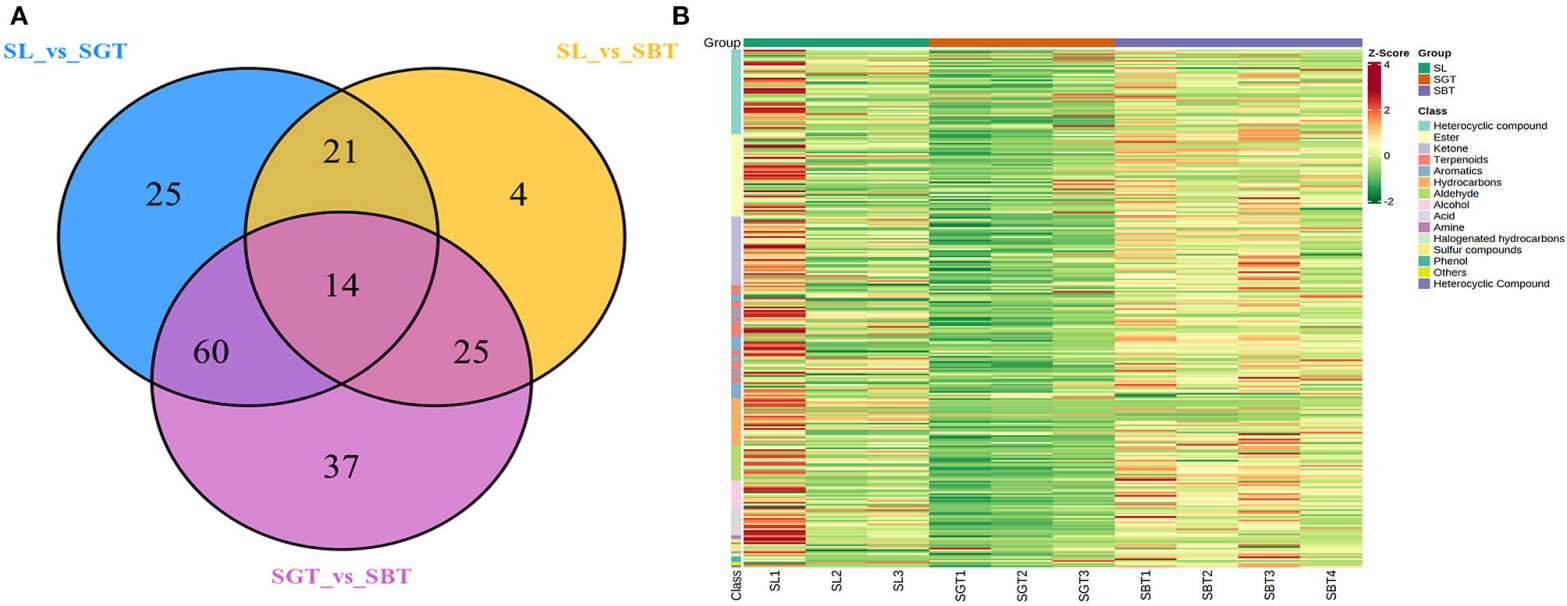
Gas chromatography-mass spectrometry (GC-MS) analysis of SL, SGT, and SBT. **(A)** Venn plot. **(B)** Heat map.

The information of 295 volatile metabolites was given in Supplementary Table S1. The volatile components in the tea are mainly composed of esters (47), heterocyclic compounds (49), ketones (39), hydrocarbons (27), terpenes (32), acids (14), aldehydes (20), aromatic hydrocarbons (33), alcohols (17), and others (17). In the Venn plot (Figure 4A), 14 metabolites were detected in all the samples, 25, 4, and 37 metabolites were specific to SL, SGT, and SBT, respectively; 21, 60, and 25 metabolites were specific to SL and SGT, SL and SBT, and SGT and SBT, respectively. These results indicated that different processing methods may be have a great impact on the volatile chemical composition of sea buckthorn leaves tea process. Aroma components in tea are mainly composed of esters and alcohols and the unique aroma of each tea is mainly determined by the difference of aromatic components and combination concentration (19). The average content data of volatile metabolites of different categories detected in SL, SGT, and SBT were analyzed by heat map (Figure 4B). Comparing with SL, the contents of aldehyde, aromatic, ester, and heterocyclic compounds in SBT increased after fermentation from 11.98 to 14.85%, from 5.6 to 7.57%, from 18.02 to 24.97%, and from 9.74 to 10.3%, respectively. The aroma varieties of sea buckthorn leaves tea increased significantly after heat treatment and fermentation compared with the original leaves, which provided a method for the processing of sea buckthorn leaves.

**Figure 5:**
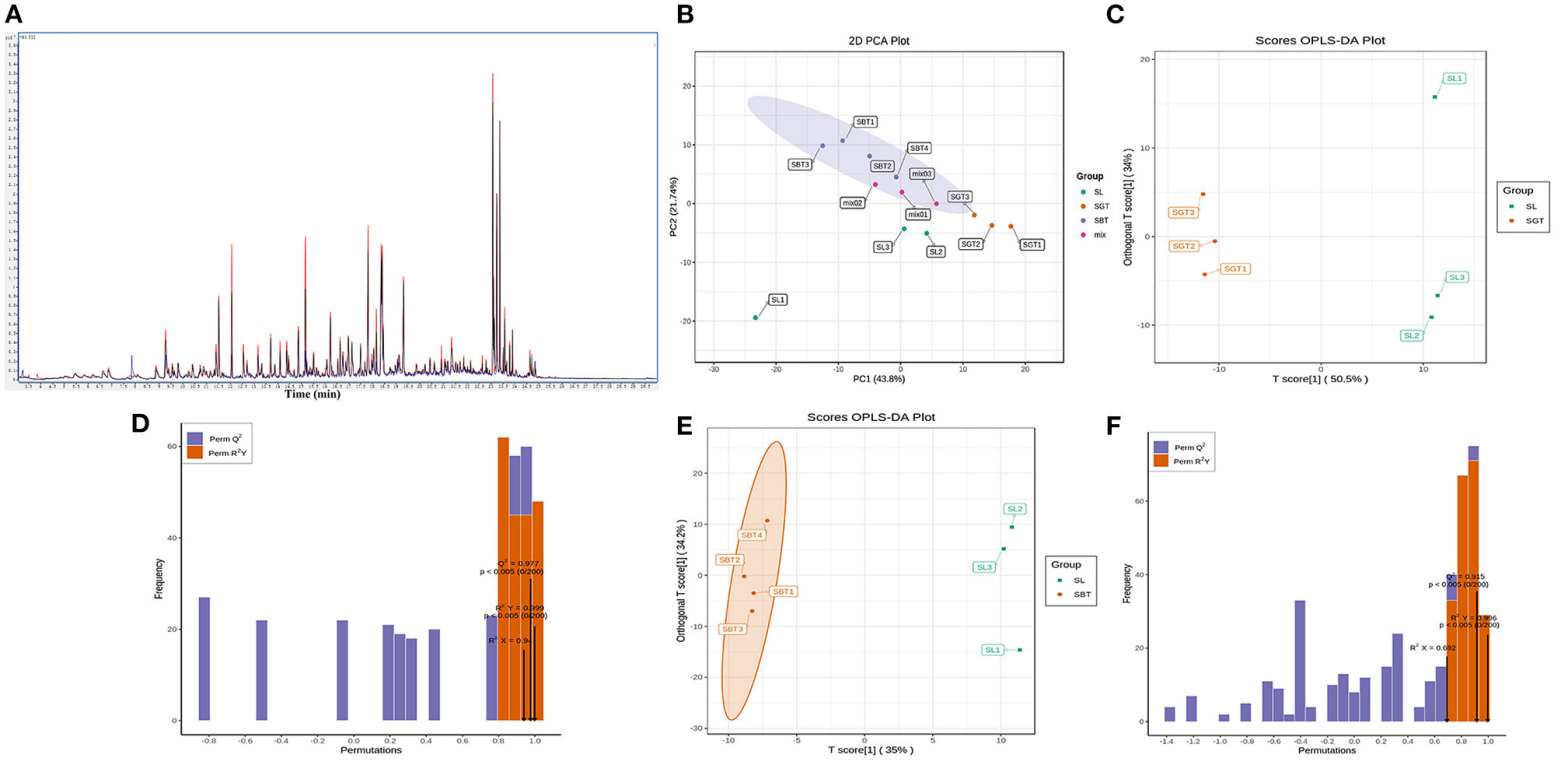
The multivariate statistical analyses of SL, SGT, and SBT from GC-MS. **(A)** Total ion chromatogram (TIC) overlap pattern was detected by quality spectrum of quality control samples in GC-MS. **(B)** PCA score plot. **(C)** SL vs. SGT OPLS-DA score plot. **(D)** SL vs. SGT OPLS-DA permutation plot. **(E)** SL vs. SBT OPLS-DA score plot. **(F)** SL vs. SBT OPLS-DA permutation plot.

**Figure 6:**
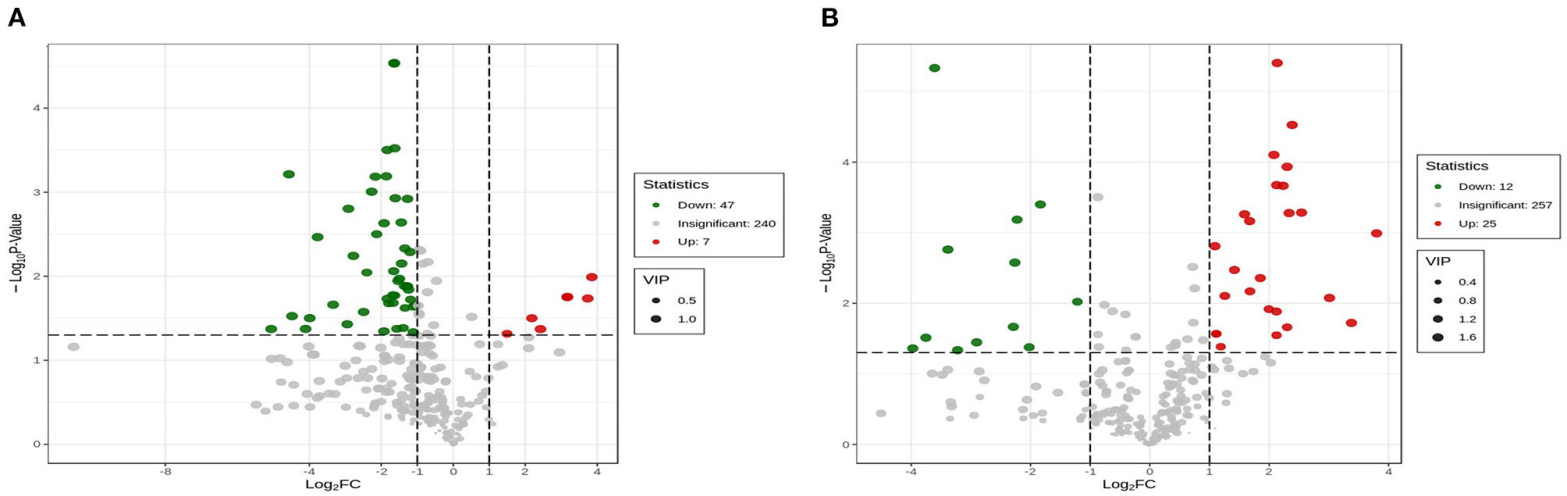
Volcano Plot of SL, SGT, and SBT of GC-MS. **(A)** SL vs. SBT and **(B)** SL vs. SGT.

The dendrogram of the samples was divided into three types; the PCA (Figure 5B) and OPLS-DA showed significantly different from SGT and SBT (Figures 5C,E). Cross-validation with 200 permutation tests showed that the OPLS-DA model is reliable, the R^2^X, R^2^Y, and Q^2^ of SL and SGT were, respectively, 0.89, 0.986, and 0.965 (Figure 5D), and the R^2^X, R^2^Y, and Q^2^ of SL and SBT were, respectively, 0.94, 0.999, and 0.977 (Figure 5F). Based on OPLS-DA results, VIP value > 1 with FC > 2 or FC < 0.5, *p*-value < 0.05, was used as the screening condition; 7 metabolites were significantly upregulated between SL and SGT, while 25 metabolites were significantly upregulated between SL and SBT (Figures 6A,B). The Maillard reaction is a classical reaction resulting from the food heating process, increasing of acids, aldehydes, ketones, and methylpyrazines (20). The content of 2,3,5-trimethyl-6-ethylpyrazine, 2,3-dimethyl-5-ethylpyrazine, 2, 5-dimethyl-3-ethylpyrazine, 3, 5-diethylmethylpyrazine, methyl pelargonate, phenol, (+)-2-bornanone, 3-hexen-1-ol benzoate, methyl benzoate, and methyl decanoate remarkably increased in SGT, which impart desirable flavors to the material that could produce darker color, distinctive flavor of nutty toasted sweet aroma of SGT. The unique flavor and texture of the fermented SBT were contributed by the presence of microorganisms and their byproduct produced during fermentation. Hexenal, (Z)−9-hexadecenoate methyl ester, vitispirane, phthalic acid, butyl hex-2-yn-4-yl ester, 3-methyl-2-butenoic acid, cyclobutyl ester, isoelemicin, methyl nonanoate, 3-furaldehyde, ethyl benzoate, and benzoic acid dramatically improved in SBT, which impart desirable flavors of pleasant fruity sweetness aroma. In a nutshell, during thermal treatment and fermentation process, high amounts of Maillard reaction products (MRPs) including melanoidins were formed, which were contributed to flavor and color of sea buckthorn leaves tea. Instead, the fermentation process of sea buckthorn leaves makes the polyphenols susceptible to enzymatic oxidation, allowing the conversion of catechins into polymeric compounds, such as theaflavins and thearubigins, which confer the characteristic aroma and color, typical of the black tea (21). Together, these results suggested that different processes of sea buckthorn leaves have significant effects on the non-volatile and volatile metabolites, while the fermentation process has the greatest impact on the metabolic profiles of its volatile components.

### Antioxidant Activities

**Figure 7:**
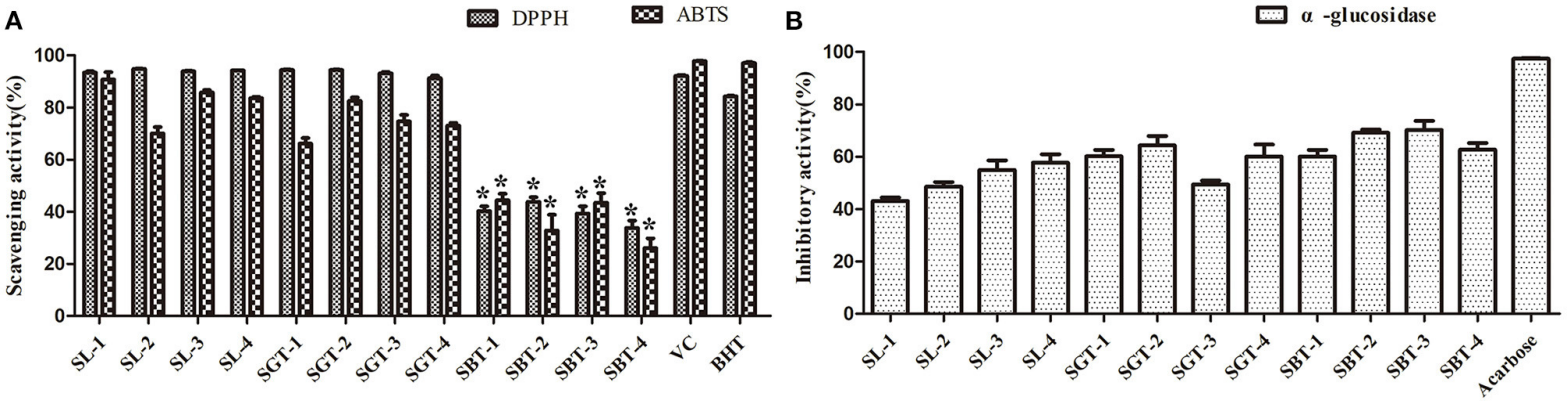
2,2-diphenyl-1-picrylhydrazyl (DPPH), 2,2'-azino-bis(3-ethylbenzothiazoline-6-sulfonic acid) diammonium salt (ABTS), and α-glucosidase inhibitory activities of sea buckthorn leaves tea. **(A)** Antioxidant activities. **(B)** α-glucosidase inhibitory activities. Data are presented as mean ± SD (*p < .05, *n* = 3).

**Figure 8:**
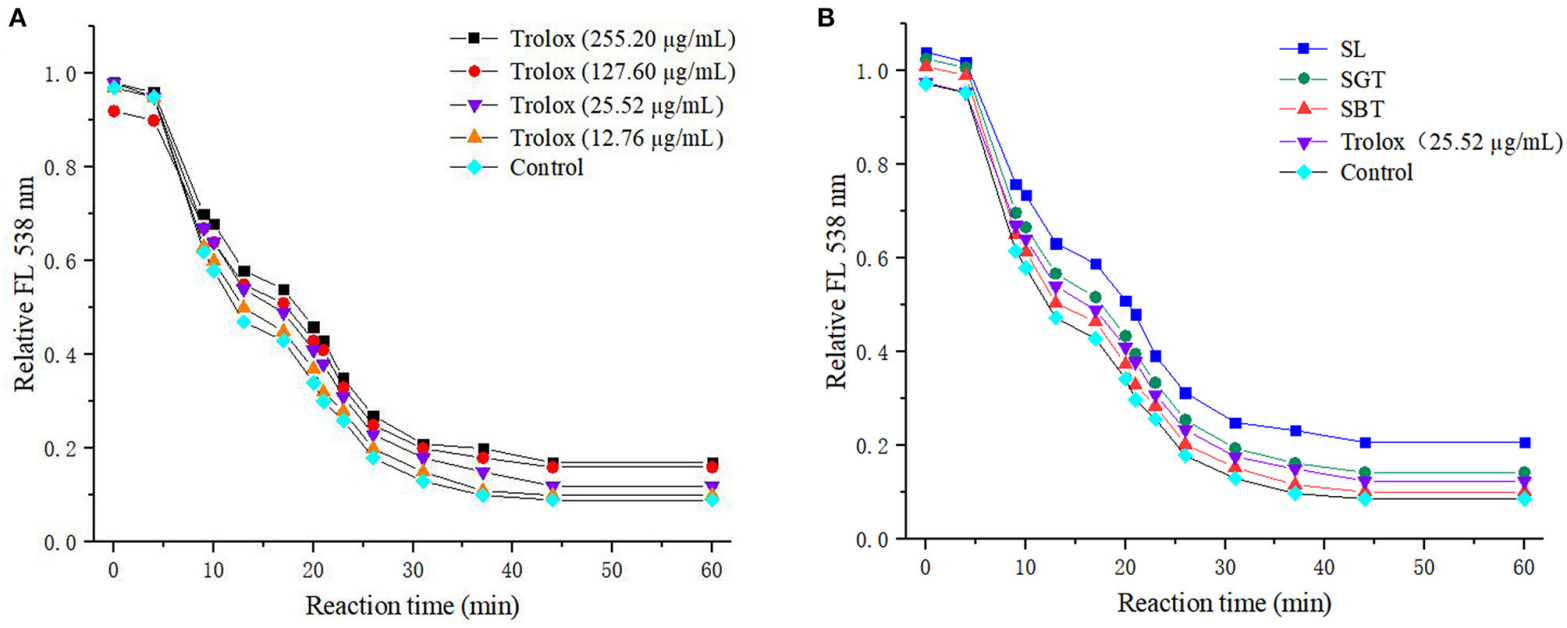
Fluorescence decay curves induced by AAPH in the presence of Trolox **(A)** and sea buckthorn leaves tea **(B)**.

Phenolic compounds are a safe and effective source of antioxidant, removing excess free radicals to promote human health (22). According to the gallic acid standard curve to calculate (y = 0.0655x−0.0473, R^2^ = 0.9993), total polyphenol contents of SL, SGT, and SBT were 103.35–109.14, 90.39–103.55, and 49.86–55.74 mg gallic acid equivalent (GAE)/g, respectively (Table 2). Flavonoids from natural sources have significant biological activities, such as antioxidation, antimutation, anticancer, and vascular protection (23). According to the calculation of rutin standard curve (y = 0.0059x + 0.1798, R^2^ = 0.9992), total flavonoid content of SL, SGT, and SBT were 24.57–34.22, 26.66–33.34, and 13.35–16.86 mg Rutin/g, respectively (Table 2). After fermentation, the content of total polyphenols and flavonoids decreased sharply in SBT. Recent studies have shown that appropriate intake of foods with antioxidant effects could reduce oxidative stress damage caused by free radicals. *In-vitro* antioxidant screening methods are often used to evaluate antioxidant capacity due to their high throughput and low cost (24). The antioxidant activities of radical scavenging on 2,2-diphenyl-1-picrylhydrazyl (DPPH) and 2,2'-azino-bis(3-ethylbenzothiazoline-6-sulfonic acid) diammonium salt (ABTS) were evaluated. The DPPH radical and ABTS radical scavenging rates are shown in Figure 7A. At the concentration of 0.1 mg/ml, the DPPH radical scavenging activity of SL, SGT, and SBT was 93.42 to 94.80%, 91.24 to 94.44%, and 33.83 to 43.84%, respectively. The ABTS radical scavenging activity of SL, SGT, and SBT was 70.02 to 90.77%, 66.15 to 82.55%, and 26.08 to 44.38%, respectively. There was a clear trend of decreasing of the antioxidant activities of SBT compared with SL and SGT (*p* < 0.05). The Pearson product-moment correlation coefficient was used to determine the relationship between TFC, TPC, and antioxidant activities, TFC and DPPH (*r* = 0.94), TPC and DPPH (*r* = 0.98), TFC and ABTS (*r* = 0.85), and TPC and ABTS (*r* = 0.92). The scavenging ability of DPPH and ABTS was highly positive correlated with the contents of total polyphenol and flavonoids in SL and SGT. Several studies have documented the main components of sea buckthorn leaves, gallic acid, rutin, quercetin-3-galactoside, quercetin-3-glucoside, myricetin, quercetin, kaempferol, and isorhamnetin, which are responsible for great antioxidant activity (25), which were also detected in SL, SGT, and SBT (Table 1). Oxygen radical absorbance capacity (ORAC) is an important evaluation standard for the total antioxidant capacity of functional food. The ORAC assays were used to evaluate the antioxidant activity of sea buckthorn leaves tea and the results are shown in Figure 8. The areas under the curve of fluorescence attenuation indicate the protective effect of antioxidants. According to the changes of fluorescence intensity in the control group, AAPH degrades in the solution and produces free radicals, which react with luciferase and weaken the fluorescence signal. As an antioxidant, the protective effect of Trolox on fluorescein fluorescence intensity was proportional to its concentration (Figure 8A). When the concentration was 0.1 mg/ml, SL, SGT, and SBT could inhibit the reaction of free radicals produced by AAPH and fluorescein, prolonging the weakening time of fluorescence signal. Trolox was selected as a positive control in ORAC assays. The antioxidant activities of ORAC assays were SL > SGT > Trolox (25.52 μg/ml) > SBT (Figure 8B). A higher protective capacity was observed for SL than for SGT and SBT in the fluorescence decay curves. Consistent with the behavior observed in the DPPH and ABTS assays, fermentation process of sea buckthorn leaves negatively affected the antioxidant capacity. A comparison of all the results reveals that sea buckthorn leaves tea was rich in polyphenol and flavonoids and could be used as a new dietary supplement with high antioxidant content.

**Table 2:** Total flavonoid content (TFC), total polyphenol content (TPC), antioxidant, and α-glucosidase inhibitory activities of sea buckthorn leaves (SL), sea buckthorn leaves green tea (SGT), and sea buckthorn leaves black tea (SBT) (*n* = 3).

**No**.	**TFC($\overset{-}{X}$ ±SD, mg Rutin/g)**	**TPC($\overset{-}{X}$ ±SD, mg GAE/g)**	**DPPH scavenging activities ($\overset{-}{X}$% ±SD)**	**ABTS scavenging activities ($\overset{-}{X}$% ±SD)**	***α*** **-glucosidase inhibitory activities ($\overset{-}{X}$% ±SD)**
SL-1	24.57 ± 0.033 ^a^	1091.38 ± 0.021 ^a^	93.42% ± 0.005 ^a^	90.77% ± 0.028 ^a^	43.07% ± 0.013 ^a^
SL-2	29.99 ± 0.025 ^a^	1067.89 ± 0.018 ^a^	94.80% ± 0.002 ^a^	70.02% ± 0.025 ^a^	48.56% ± 0.017 ^a^
SL-3	33.09 ± 0.026 ^a^	1041.37 ± 0.013 ^a^	93.88%±0.003 ^a^	85.76% ± 0.010 ^a^	54.92% ± 0.037 ^a^
SL-4	34.83 ± 0.025 ^a^	1033.46 ± 0.013 ^a^	94.24% ± 0.001 ^a^	83.59% ± 0.005 ^a^	57.67% ± 0.032 ^a^
SGT-1	33.34 ± 0.032 ^a^	1035.45 ± 0.017 ^a^	94.44% ± 0.002 ^a^	66.15% ± 0.021 ^b^	60.17% ± 0.024 ^a^
SGT-2	29.50 ± 0.036 ^a^	990.15 ± 0.038 ^a^	94.42% ± 0.002 ^a^	82.55% ± 0.014 ^b^	64.34% ± 0.035 ^a^
SGT-3	29.02 ± 0.031 ^a^	990.15 ± 0.030 ^a^	93.14% ± 0.005 ^a^	74.67% ± 0.025 ^b^	49.35% ± 0.015 ^a^
SGT-4	26.66 ± 0.030 ^a^	903.94 ± 0.058 ^a^	91.24% ± 0.010 ^a^	73.09% ± 0.010 ^b^	60.11% ± 0.045 ^a^
SBT-1	15.69 ± 0.011 ^b^	501.82 ± 0.020 ^b^	40.26% ± 0.018 ^b^	44.38% ± 0.025 ^c^	60.11% ± 0.025 ^a^
SBT-2	16.87 ± 0.011 ^b^	535.66 ± 0.006 ^b^	43.84% ± 0.017 ^b^	32.81% ± 0.061 ^c^	69.08% ± 0.012 ^a^
SBT-3	15.33 ± 0.010 ^b^	498.56 ± 0.006 ^b^	39.33% ± 0.027 ^b^	43.53% ± 0.036 ^c^	70.16% ± 0.035 ^a^
SBT-4	13.35 ± 0.036 ^b^	557.39 ± 0.022 ^b^	33.83% ± 0.028 ^b^	26.08% ± 0.038 ^c^	62.65% ± 0.026 ^a^

*Values in the same column labeled with different superscript letters differ significantly (p < 0.05)*.

### α-Glucosidase Inhibitory Activities

Diabetes is a chronic metabolic disorder often associated with complications, which has become one of the most harmful diseases to human health in the world; the α-glucosidase inhibitors are oral hypoglycemic drugs with definite efficacy for type 2 diabetes (26). At the concentration of 4 mg/ml, the extracts of sea buckthorn leaves tea showed moderate α-glucosidase inhibition ability compared to acarbose (0.97 mg/ml) (Figure 7B). The α-glucosidase inhibitory activities of SL, SGT, and SBT were 43.07–57.67%, 49.35–64.34%, and 60.11–70.16%, respectively. No significant difference in α-glucosidase inhibitory activities was observed between SL and SGT, SL and SBT, and SGT and SBT (*p* >.05), respectively. It is of great significance for comprehensive development and utilization of sea buckthorn leaves in food and medicine field.

## Conclusion

The phytochemical constituents in sea buckthorn leaves tea have been investigated via LC-MS, HS-SPME-GC-MS, and HPLC fingerprint combined with the multivariate statistical analysis. Our findings clearly indicated that the components of sea buckthorn leaves varied greatly with different processing, forming their own unique quality characteristics of sea buckthorn leaves tea. Moreover, the extracts of sea buckthorn leaves tea have marked antioxidant and α-glucosidase inhibitory activities *in vitro*, highlighting the potential usefulness of sea buckthorn leaves tea that could be developed as a natural health food. These investigations provided theoretical references for further development and utilization of sea buckthorn leaves resources.

## Data Availability Statement

The original contributions presented in the study are included in the article/Supplementary Material, further inquiries can be directed to the corresponding author.

## Author Contributions

NW contributed to conceptualization, methodology, investigation, assays, and writing—original draft preparation. ZY contributed to supervision, project administration, writing—review and editing, and funding acquisition. XW, YG, SL, YL, and YS contributed to visualization. All the authors have read and agreed to the published version of the manuscript. All the authors contributed to the article and approved the submitted version of the manuscript.

## Funding

This study received funding from the Key Project of NMPA Key Laboratory for Quality Control of Traditional Chinese Medicine (2020GSMPA-KL11), the Natural Science Foundation of Gansu Province (20JR5RA311), and the Fundamental Research Funds for the Central Universities (lzujbky-2021-kb40, lzujbky-2020-46).

## Conflict of Interest

The authors declare that the research was conducted in the absence of any commercial or financial relationships that could be construed as a potential conflict of interest.

## Publisher's Note

All claims expressed in this article are solely those of the authors and do not necessarily represent those of their affiliated organizations, or those of the publisher, the editors and the reviewers. Any product that may be evaluated in this article, or claim that may be made by its manufacturer, is not guaranteed or endorsed by the publisher.
