# Supplementary material for: Identification and Characterization of the Bioactive Polyphenols and Volatile Compounds in Sea Buckthorn Leaves Tea Together With Antioxidant and α-Glucosidase Inhibitory Activities
Source: Front Nutr. 2022 Apr 29;9:890486. doi: 10.3389/fnut.2022.890486 (PMC9100590; doi:10.3389/fnut.2022.890486)
Supplement: Supplementary file 1 [file Data_Sheet_1.docx]

**Supplementary Data**

**Identification and Characterization of the Bioactive Polyphenols and Volatile Compounds from Seabuckthorn Leaves Tea together with Antioxidant and α-glucosidase Inhibitory Activities**

Ningning Wang ^a^, Xiufeng Wen ^b^, Yan Gao ^b^, Shunguang Lu ^b^, Yimeng Li ^a,c^, Yanbin Shi ^a,c^, Zhigang Yang ^a,c*^

a School of Pharmacy, Lanzhou University, Lanzhou, P.R. China.

b Seabuckthorn Development and Management Center of the Ministry of Water Resources, Beijing, P.R. China.

c Collaborative Innovation Center for Northwestern Chinese Medicine, Lanzhou University, Lanzhou P.R. China.

* To whom correspondence should be addressed.

Correspondence: E-mail: yangzg@lzu.edu.cn; Tel.:+86-931-8915202

**Table S1** Information of seabuckthorn leaves tea samples

| Sample | Type | Collection time |
| --- | --- | --- |
| SL-1 | seabuckthorn leaves | 2021.9 |
| SL-2 | seabuckthorn leaves | 2021.9 |
| SL-3 | seabuckthorn leaves | 2021.9 |
| SL-4 | seabuckthorn leaves | 2021.9 |
| SGT-1 | seabuckthorn leaves green tea | 2021.9 |
| SGT-2 | seabuckthorn leaves green tea | 2021.9 |
| SGT-3 | seabuckthorn leaves green tea | 2021.9 |
| SGT-4 | seabuckthorn leaves green tea | 2021.9 |
| SBT-1 | seabuckthorn leaves black tea | 2021.9 |
| SBT-2 | seabuckthorn leaves black tea | 2021.9 |
| SBT-3 | seabuckthorn leaves black tea | 2021.9 |
| SBT-4 | seabuckthorn leaves black tea | 2021.9 |

**Table S2** 295 volatile metabolites were identified of seabuckthorn leaves tea

| No. | Molecular Weight (Da) | RI | NIST_RI | Compounds | Class I |
| --- | --- | --- | --- | --- | --- |
| 1 | 1.12E+02 | 9.61E+02 | 9.67E+02 | Benzaldehyde-d6 | Aromatics |
| 2 | 2.02E+02 | 1.26E+03 | 1.52E+03 | Naphthalene, 1,2,3,4-tetrahydro-1,6-dimethyl-4-(1-methylethyl)-, (1S-cis)- | Terpenoids |
| 3 | 1.96E+02 | 1.16E+03 | 1.41E+03 | 2-Furancarboxylic acid, hexyl ester | Ester |
| 4 | 1.94E+02 | 1.45E+03 | 1.45E+03 | 5,9-Undecadien-2-one, 6,10-dimethyl-, (E)- | Ketone |
| 5 | 1.92E+02 | 1.44E+03 | 1.39E+03 | Benzoic acid, 2-methylbutyl ester | Ester |
| 6 | 1.88E+02 | 1.68E+03 | 1.68E+03 | 1(3H)-Isobenzofuranone, 3-butylidene- | Heterocyclic compound |
| 7 | 1.78E+02 | 1.48E+03 | 1.42E+03 | 1-(3-Ethoxyphenyl)acetone | Ketone |
| 8 | 1.72E+02 | 1.24E+03 | 1.20E+03 | Ethyl 4-(ethyloxy)-2-oxobut-3-enoate | Ester |
| 9 | 1.70E+02 | 9.83E+02 | 1.23E+03 | 1,3,3-trimethyl-2-Oxabicyclo[2.2.2]octan-6-ol | Terpenoids |
| 10 | 1.68E+02 | 1.24E+03 | 1.18E+03 | 2-Methylisoborneol | Terpenoids |
| 11 | 1.52E+02 | 9.10E+02 | 1.12E+03 | (+)-2-Bornanone | Terpenoids |
| 12 | 1.52E+02 | 1.15E+03 | 1.16E+03 | 6-Octenal, 7-methyl-3-methylene- | Aldehyde |
| 13 | 1.52E+02 | 1.22E+03 | 1.25E+03 | 2,6,6-trimethyl-1-Cyclohexene-1-carboxaldehyde | Terpenoids |
| 14 | 1.50E+02 | 1.31E+03 | 1.16E+03 | Pyrazine, 2,3-diethyl-5-methyl- | Heterocyclic compound |
| 15 | 1.50E+02 | 1.15E+03 | 1.16E+03 | 2,3,5-Trimethyl-6-ethylpyrazine | Heterocyclic compound |
| 16 | 1.50E+02 | 1.18E+03 | 1.19E+03 | Benzoic acid, ethyl ester | Ester |
| 17 | 1.46E+02 | 1.27E+03 | 1.28E+03 | BenzeneacetAldehyde, .alpha.-ethylidene- | Aldehyde |
| 18 | 1.42E+02 | 1.30E+03 | 1.31E+03 | Naphthalene, 1-methyl- | Aromatics |
| 19 | 1.38E+02 | 1.13E+03 | 1.10E+03 | 3-Acetyl-2,5-dimethyl furan | Heterocyclic compound |
| 20 | 1.36E+02 | 1.08E+03 | 1.08E+03 | 2,3-Dimethyl-5-ethylpyrazine | Heterocyclic compound |
| 21 | 1.36E+02 | 1.09E+03 | 1.02E+03 | Pyrazine, (2-methylpropyl)- | Heterocyclic compound |
| 22 | 1.36E+02 | 1.13E+03 | 1.11E+03 | Benzene, 1-ethyl-4-methoxy- | Aromatics |
| 23 | 1.34E+02 | 1.03E+03 | 1.13E+03 | Benzene, 1,2,3,5-tetramethyl- | Aromatics |
| 24 | 1.32E+02 | 1.09E+03 | 1.07E+03 | Indan, 1-methyl- | Aromatics |
| 25 | 1.25E+02 | 1.19E+03 | 1.08E+03 | 2-Fluoro-5-methylaniline | Amine |
| 26 | 1.24E+02 | 8.83E+02 | 9.96E+02 | Ethanone, 1-(2-methyl-1-cyclopenten-1-yl)- | Ketone |
| 27 | 1.16E+02 | 9.57E+02 | 9.11E+02 | 1-Hexanol, 4-methyl- | Alcohol |
| 28 | 4.01E+02 | 1.63E+03 | - | 4-fluoro-3-nitro-benzoic acid, 2,4-bis(1,1-dimethylpropyl)phenyl ester | Ester |
| 29 | 3.55E+02 | 1.13E+03 | - | Cobalt, bis(.eta.-5-piperidinylcyclopentadienyl)- | Heterocyclic compound |
| 30 | 3.48E+02 | 2.90E+03 | 2.47E+03 | Sulfurous acid, 2-ethylhexyl undecyl ester | Ester |
| 31 | 3.38E+02 | 1.84E+03 | 2.17E+03 | Phytol, acetate | Alcohol |
| 32 | 3.20E+02 | 1.86E+03 | 2.09E+03 | Phthalic acid, hept-4-yl isobutyl ester | Ester |
| 33 | 3.20E+02 | 1.96E+03 | 2.13E+03 | Phthalic acid, butyl hept-4-yl ester | Ester |
| 34 | 3.10E+02 | 2.02E+03 | 2.19E+03 | E-6-Octadecen-1-ol acetate | Ester |
| 35 | 3.02E+02 | 1.90E+03 | 2.19E+03 | Phthalic acid, butyl hex-2-yn-4-yl ester | Ester |
| 36 | 2.98E+02 | 2.12E+03 | 2.15E+03 | Methyl stearate | Ester |
| 37 | 2.96E+02 | 2.11E+03 | 2.11E+03 | Phytol | Terpenoids |
| 38 | 2.96E+02 | 1.95E+03 | 1.95E+03 | Isophytol | Terpenoids |
| 39 | 2.96E+02 | 2.10E+03 | 2.09E+03 | 9-Octadecenoic acid (Z)-, methyl ester | Ester |
| 40 | 2.94E+02 | 1.74E+03 | 1.91E+03 | 3,7,11,15-tetramethyl-1-Hexadecyn-3-ol | Alcohol |
| 41 | 2.86E+02 | 1.59E+03 | 1.61E+03 | 2,2,4-Trimethyl-1,3-pentanediol diisobutyrate | Ester |
| 42 | 2.84E+02 | 1.99E+03 | 1.99E+03 | Hexadecanoic acid, ethyl ester | Ester |
| 43 | 2.84E+02 | 2.02E+03 | 1.91E+03 | Hexadecanoic acid, 15-methyl-, methyl ester | Ester |
| 44 | 2.82E+02 | 2.30E+03 | 2.00E+03 | Eicosane | Hydrocarbons |
| 45 | 2.82E+02 | 1.75E+03 | 1.75E+03 | Hexadecane, 2,6,11,15-tetramethyl- | Hydrocarbons |
| 46 | 2.82E+02 | 1.81E+03 | 1.75E+03 | Hexadecane, 2,6,10,14-tetramethyl- | Hydrocarbons |
| 47 | 2.82E+02 | 2.17E+03 | 2.14E+03 | cis-Vaccenic Acid | Acid |
| 48 | 2.81E+02 | 1.70E+03 | 1.76E+03 | N-(2,6-Diethylphenyl)-1,1,1-trifluoromethane sulfonamide | Amine |
| 49 | 2.76E+02 | 1.91E+03 | 2.08E+03 | 7,9-Di-tert-butyl-1-oxaspiro(4,5)deca-6,9-diene-2,8-dione | Heterocyclic compound |
| 50 | 2.70E+02 | 1.92E+03 | 1.93E+03 | Hexadecanoic acid, methyl ester | Ester |
| 51 | 2.68E+02 | 1.84E+03 | 1.77E+03 | 2-Pentadecanone, 6,10,14-trimethyl- | Ketone |
| 52 | 2.68E+02 | 1.90E+03 | 1.90E+03 | 9-Hexadecenoic acid, methyl ester, (Z)- | Ester |
| 53 | 2.62E+02 | 1.91E+03 | 2.02E+03 | 5,9,13-Pentadecatrien-2-one, 6,10,14-trimethyl-, (E,E)- | Ketone |
| 54 | 2.61E+02 | 1.53E+03 | 1.55E+03 | l-Alanine, N-(2,3,4-trifluorobenzoyl)-, methyl ester | Ester |
| 55 | 2.58E+02 | 1.85E+03 | 1.85E+03 | 1,3,4,6,7,8-hexahydro-4,6,6,7,8,8-hexamethyl-Cyclopenta[g]-2-benzopyran | Heterocyclic compound |
| 56 | 2.56E+02 | 1.79E+03 | 1.69E+03 | Tetradecanoic acid, ethyl ester | Ester |
| 57 | 2.56E+02 | 1.82E+03 | 1.78E+03 | Pentadecanoic acid, methyl ester | Ester |
| 58 | 2.54E+02 | 1.77E+03 | 1.77E+03 | Heptadecane, 2-methyl- | Hydrocarbons |
| 59 | 2.54E+02 | 1.77E+03 | 1.75E+03 | Heptadecane, 3-methyl- | Hydrocarbons |
| 60 | 2.54E+02 | 1.94E+03 | 1.95E+03 | Palmitoleic Acid | Acid |
| 61 | 2.48E+02 | 1.40E+03 | 1.60E+03 | 1,3-Dimethyl-5-n-hexyladamantane | Hydrocarbons |
| 62 | 2.42E+02 | 1.68E+03 | 1.50E+03 | 2-hexyl-1-Decanol | Alcohol |
| 63 | 2.42E+02 | 1.72E+03 | 1.72E+03 | Methyl tetradecanoate | Ester |
| 64 | 2.42E+02 | 1.86E+03 | 1.87E+03 | Pentadecanoic Acid | Acid |
| 65 | 2.42E+02 | 1.09E+03 | 1.09E+03 | Ethyl 2-(5-methyl-5-vinyltetrahydrofuran-2-yl)propan-2-yl carbonate | Heterocyclic compound |
| 66 | 2.40E+02 | 1.70E+03 | 1.70E+03 | Heptadecane | Hydrocarbons |
| 67 | 2.40E+02 | 1.82E+03 | 1.81E+03 | Hexadecanal | Aldehyde |
| 68 | 2.36E+02 | 1.51E+03 | - | 8-(2-Acetyloxiran-2-yl)-6,6-dimethylocta-3,4-dien-2-one | Ketone |
| 69 | 2.34E+02 | 1.76E+03 | 1.62E+03 | 3,5-DITERTBUTYLSALICYLAldehyde | Aldehyde |
| 70 | 2.34E+02 | 1.71E+03 | 1.74E+03 | Benzoic acid, 2-ethylhexyl ester | Ester |
| 71 | 2.32E+02 | 1.89E+03 | 1.66E+03 | 1,8,11,14-Heptadecatetraene, (Z,Z,Z)- | Hydrocarbons |
| 72 | 2.32E+02 | 1.62E+03 | 1.88E+03 | 1,1'-(6-methoxy-2,5-benzofurandiyl)bis-Ethanone | Heterocyclic compound |
| 73 | 2.30E+02 | 1.31E+03 | 1.31E+03 | 2,3,4-Trifluorobenzoic acid, cyclobutyl ester | Ester |
| 74 | 2.28E+02 | 1.59E+03 | 1.59E+03 | Dodecanoic acid, ethyl ester | Ester |
| 75 | 2.28E+02 | 1.76E+03 | 1.77E+03 | Tetradecanoic Acid | Acid |
| 76 | 2.28E+02 | 1.53E+03 | 1.59E+03 | Fumaric acid, ethyl tetrahydrofurfuryl ester | Ester |
| 77 | 2.26E+02 | 1.60E+03 | 1.60E+03 | Hexadecane | Hydrocarbons |
| 78 | 2.26E+02 | 1.46E+03 | 1.42E+03 | 2,6,10-Trimethyltridecane | Hydrocarbons |
| 79 | 2.26E+02 | 1.67E+03 | 1.57E+03 | 3-methyl-Pentadecane | Hydrocarbons |
| 80 | 2.26E+02 | 1.48E+03 | 1.40E+03 | 4-Heptanone, 5,5-diethyl-2,2,3,3-tetramethyl- | Ketone |
| 81 | 2.24E+02 | 1.41E+03 | 1.36E+03 | 2,4a-Epidioxy-5,6,7,8-tetrahydro-2,5,5,8a-tetramethyl-2H-1-benzopyran | Heterocyclic compound |
| 82 | 2.22E+02 | 1.16E+03 | - | Nerolidol 1 | Terpenoids |
| 83 | 2.22E+02 | 1.66E+03 | 1.67E+03 | 2-Hydroxy-2,4,4-trimethyl-3-(3-methylbuta-1,3-dienyl)cyclohexanone | Ketone |
| 84 | 2.22E+02 | 1.59E+03 | 1.59E+03 | Diethyl Phthalate | Ester |
| 85 | 2.20E+02 | 1.39E+03 | 1.41E+03 | (1R,2R,4S,6S,7S,8S)-8-Isopropyl-1-methyl-3-methylenetricyclo[4.4.0.02,7]decan-4-ol | Terpenoids |
| 86 | 2.20E+02 | 1.50E+03 | 1.72E+03 | p-Methoxyheptanophenone | Ketone |
| 87 | 2.20E+02 | 1.50E+03 | 1.66E+03 | 2H-1-Benzopyran, 6,7-dimethoxy-2,2-dimethyl- | Aromatics |
| 88 | 2.18E+02 | 1.39E+03 | 1.46E+03 | Benzene, [1-[[1-(1-methylethyl)-3-butenyl]oxy]ethyl]-, [S-(R*,R*)]- | Aromatics |
| 89 | 2.18E+02 | 1.71E+03 | 1.59E+03 | 1,2,3,4,5,6-Hexahydro-1,1,5,5-tetramethyl-2,4a-methanonaphthalen-7(4aH)-one | Terpenoids |
| 90 | 2.18E+02 | 1.70E+03 | 1.90E+03 | 5H-Oxazolo[3,2-a]pyridine-8-carbonitrile, 6-Ethyl-2,3-dihydro-2,7-dimethyl-5-oxo- | Heterocyclic compound |
| 91 | 2.17E+02 | 1.46E+03 | 1.68E+03 | (1R,4S,9aS)-1-Methyl-4-((Z)-pent-2-en-4-yn-1-yl)octahydro-1H-quinolizine | Heterocyclic compound |
| 92 | 2.14E+02 | 1.52E+03 | 1.51E+03 | Dodecanoic acid, methyl ester | Ester |
| 93 | 2.12E+02 | 1.56E+03 | 1.47E+03 | 3-methyl-Tetradecane | Hydrocarbons |
| 94 | 2.12E+02 | 1.50E+03 | 1.50E+03 | Pentadecane | Hydrocarbons |
| 95 | 2.12E+02 | 1.16E+03 | 1.42E+03 | 1,3:4,5-di-O-(ethylboranediyl)-2-deoxy-Ribitol | Heterocyclic compound |
| 96 | 2.10E+02 | 1.68E+03 | 1.66E+03 | 2,2',5,5'-tetramethyl-1,1'-Biphenyl | Aromatics |
| 97 | 2.10E+02 | 1.71E+03 | 1.74E+03 | 9,9-dimethyl-Xanthene | Aromatics |
| 98 | 2.08E+02 | 1.48E+03 | 1.61E+03 | 3-Buten-2-one, 4-(2,2,6-trimethyl-7-oxabicyclo[4.1.0]hept-1-yl)- | Terpenoids |
| 99 | 2.08E+02 | 1.61E+03 | 1.70E+03 | 3-Hydroxy-7,8-dihydro-.beta.-ionol | Terpenoids |
| 100 | 2.08E+02 | 1.42E+03 | 1.48E+03 | 6-Methyl-6-(5-methylfuran-2-yl)heptan-2-one | Ketone |
| 101 | 2.08E+02 | 1.63E+03 | 1.63E+03 | 4',6'-Dimethoxy-2',3'-dimethylacetophenone | Ketone |
| 102 | 2.08E+02 | 1.65E+03 | 1.65E+03 | Isoelemicin | Aromatics |
| 103 | 2.06E+02 | 1.67E+03 | 1.67E+03 | 4-Oxo-β-ionone | Terpenoids |
| 104 | 2.04E+02 | 1.56E+03 | 1.43E+03 | Elemene isomer | Terpenoids |
| 105 | 2.04E+02 | 1.50E+03 | 1.49E+03 | 1,3-Cyclohexadiene, 5-(1,5-dimethyl-4-hexenyl)-2-methyl-, [S-(R*,S*)]- | Terpenoids |
| 106 | 2.04E+02 | 1.68E+03 | 1.48E+03 | 1-Hexen, 2-(p-anisyl)-5-methyl- | Hydrocarbons |
| 107 | 2.04E+02 | 1.17E+03 | 1.37E+03 | 2-(2-butoxyethoxy)-Ethanol,acetate | Ester |
| 108 | 2.04E+02 | 1.73E+03 | 1.88E+03 | Tetrahydro-4,7,8-trimethyl-1,5-benzodiazepin-2-one | Ketone |
| 109 | 2.04E+02 | 1.46E+03 | 1.66E+03 | 1-amino-4,4-dimethyl-1-(3-pyridyl)-Pent-1-en-3-one | Heterocyclic compound |
| 110 | 2.04E+02 | 1.57E+03 | 1.57E+03 | 3-Hexen-1-ol benzoate | Ester |
| 111 | 2.03E+02 | 1.32E+03 | 1.56E+03 | (5S,8R,8aS)-8-Methyl-5-((Z)-pent-2-en-4-yn-1-yl)octahydroindolizine | Heterocyclic compound |
| 112 | 2.02E+02 | 1.57E+03 | 1.60E+03 | aR-Himachalene | Terpenoids |
| 113 | 2.00E+02 | 1.42E+03 | 1.43E+03 | Undecanoic acid, methyl ester | Ester |
| 114 | 2.00E+02 | 1.55E+03 | 1.69E+03 | Pyrimido[1,6-a]indole, 1,2,3,4-tetrahydro-2,5-dimethyl- | Heterocyclic compound |
| 115 | 1.98E+02 | 1.31E+03 | 1.29E+03 | 3,5-Dimethyldodecane | Hydrocarbons |
| 116 | 1.98E+02 | 1.40E+03 | 1.40E+03 | Tetradecane | Hydrocarbons |
| 117 | 1.96E+02 | 1.59E+03 | 1.74E+03 | Ethanone, 1-(4-hydroxy-3,5-dimethoxyphenyl)- | Ketone |
| 118 | 1.94E+02 | 1.30E+03 | 1.37E+03 | 1-Oxaspiro[4.5]dec-6-ene, 2,6,10,10-tetramethyl- | Terpenoids |
| 119 | 1.94E+02 | 1.45E+03 | 1.44E+03 | 5,9-Undecadien-2-one, 6,10-dimethyl-, (Z)- | Ketone |
| 120 | 1.94E+02 | 1.53E+03 | 1.65E+03 | 2-Butanone, 4-(4-hydroxy-3-methoxyphenyl)- | Ketone |
| 121 | 1.92E+02 | 1.42E+03 | 1.42E+03 | .alpha.-Ionone | Terpenoids |
| 122 | 1.92E+02 | 1.58E+03 | 1.43E+03 | 3,5,9-Undecatrien-2-one, 6,10-dimethyl- | Ketone |
| 123 | 1.92E+02 | 1.52E+03 | 1.52E+03 | 3-Cyclohexene-1-carboxAldehyde, 4-(4-methyl-3-pentenyl)- | Aldehyde |
| 124 | 1.92E+02 | 1.28E+03 | 1.28E+03 | vitispirane | Ester |
| 125 | 1.92E+02 | 1.42E+03 | 1.42E+03 | 2-Butanone, 4-(2,6,6-trimethyl-1,3-cyclohexadien-1-yl)- | Ketone |
| 126 | 1.92E+02 | 1.48E+03 | 1.49E+03 | trans-.beta.-Ionone | Terpenoids |
| 127 | 1.91E+02 | 1.52E+03 | - | Boron, diethyl[3-imino-2-(1-iminoethyl)butanenitrilato-N2,N3]-, (t-4)- | Others |
| 128 | 1.90E+02 | 1.38E+03 | 1.35E+03 | 2,3,5,5,8,8-hexamethyl-Cycloocta-1,3,6-triene | Hydrocarbons |
| 129 | 1.90E+02 | 1.32E+03 | 1.46E+03 | 1-(4-tert-Butylphenyl)propan-2-one | Ketone |
| 130 | 1.90E+02 | 1.38E+03 | 1.39E+03 | 2-Buten-1-one, 1-(2,6,6-trimethyl-1,3-cyclohexadien-1-yl)-, (E)- | Terpenoids |
| 131 | 1.90E+02 | 1.48E+03 | 1.49E+03 | 4-(2,6,6-Trimethylcyclohexa-1,3-dienyl)but-3-en-2-one | Ketone |
| 132 | 1.90E+02 | 1.43E+03 | 1.45E+03 | Megastigmatrienone | Ketone |
| 133 | 1.88E+02 | 1.61E+03 | 1.52E+03 | 1,1,4,5,6-Pentamethyl-2,3-dihydro-1H-indene | Aromatics |
| 134 | 1.88E+02 | 1.68E+03 | 1.68E+03 | Z-Butylidenephthalide | Hydrocarbons |
| 135 | 1.86E+02 | 1.32E+03 | 1.32E+03 | Decanoic acid, methyl ester | Ester |
| 136 | 1.84E+02 | 1.27E+03 | 1.21E+03 | Undecane, 3,5-dimethyl- | Hydrocarbons |
| 137 | 1.84E+02 | 1.32E+03 | 1.21E+03 | Undecane, 4,7-dimethyl- | Hydrocarbons |
| 138 | 1.84E+02 | 1.20E+03 | 1.30E+03 | Tridecane | Hydrocarbons |
| 139 | 1.84E+02 | 1.51E+03 | 1.69E+03 | trans-2-Dodecen-1-ol | Alcohol |
| 140 | 1.84E+02 | 1.34E+03 | 1.29E+03 | 2,2'-Isopropylidenebis(tetrahydrofuran) | Heterocyclic compound |
| 141 | 1.84E+02 | 1.39E+03 | - | Isoflurophate | Ester |
| 142 | 1.82E+02 | 1.66E+03 | 1.66E+03 | 9H-Xanthene | Aromatics |
| 143 | 1.82E+02 | 1.64E+03 | 1.64E+03 | Benzophenone | Ketone |
| 144 | 1.80E+02 | 1.54E+03 | 1.53E+03 | 2(4H)-Benzofuranone, 5,6,7,7a-tetrahydro-4,4,7a-trimethyl-, (R)- | Ester |
| 145 | 1.80E+02 | 1.52E+03 | 1.63E+03 | 1,3-Benzenediol, 5-pentyl- | Phenol |
| 146 | 1.80E+02 | 1.56E+03 | 1.70E+03 | 4',6'-Dihydroxy-2',3'-dimethylacetophenone | Ketone |
| 147 | 1.80E+02 | 1.55E+03 | 1.51E+03 | 2-Chloroethyl (2-acetylhydrazino)formate | Halogenated hydrocarbons |
| 148 | 1.76E+02 | 1.47E+03 | 1.26E+03 | 1-Isopropyl-3-tert-butylbenzene | Aromatics |
| 149 | 1.76E+02 | 1.43E+03 | 1.28E+03 | 1,3,5-Cycloheptatriene, 2,3,4,5,7,7-hexamethyl- | Hydrocarbons |
| 150 | 1.76E+02 | 1.19E+03 | 1.44E+03 | 1-Pentanone, 1-(4-methylphenyl)- | Ketone |
| 151 | 1.74E+02 | 1.23E+03 | 1.40E+03 | Naphthalene, 1,2,3,4-tetrahydro-1,4,6-trimethyl- | Aromatics |
| 152 | 1.74E+02 | 1.35E+03 | 1.35E+03 | 2,3-Dihydro-1,1,4,6-tetramethyl-1H-Indene | Aromatics |
| 153 | 1.74E+02 | 1.26E+03 | 1.45E+03 | 1,2,3,4-Tetrahydro-1,6,8-trimethyl-Naphthalene | Aromatics |
| 154 | 1.74E+02 | 1.30E+03 | 1.29E+03 | 4-(1,1-Dimethyl-2-propynyloxy)-Toluene | Aromatics |
| 155 | 1.72E+02 | 1.36E+03 | 1.37E+03 | n-Decanoic Acid | Acid |
| 156 | 1.72E+02 | 1.22E+03 | 1.22E+03 | Nonanoic acid, methyl ester | Ester |
| 157 | 1.72E+02 | 1.50E+03 | 1.47E+03 | Naphthalene, 1,2-dihydro-4,5,7-trimethyl- | Aromatics |
| 158 | 1.72E+02 | 1.36E+03 | 1.40E+03 | 1, 1, 5-Trimethyl-1, 2-dihydronaphthalene | Terpenoids |
| 159 | 1.72E+02 | 1.64E+03 | 1.37E+03 | 2-Propanone, 1-(5-methyl-3H-1,2-dithiol-3-ylidene)- | Sulfur compounds |
| 160 | 1.71E+02 | 1.29E+03 | 1.16E+03 | Cyclobutanecarboxylic acid, 2-dimethylaminoethyl ester | Ester |
| 161 | 1.70E+02 | 1.07E+03 | 1.07E+03 | 5-(3,3-Dimethyloxiran-2-Yl)-3-Methylpent-1-En-3-Ol | Acid |
| 162 | 1.70E+02 | 1.36E+03 | 1.36E+03 | 2,7-Octadiene-1,6-diol, 2,6-dimethyl- | Terpenoids |
| 163 | 1.70E+02 | 1.21E+03 | 1.22E+03 | Lilac Alcohol C | Alcohol |
| 164 | 1.70E+02 | 1.54E+03 | 1.57E+03 | 1,4,6-Trimethyl-Naphthalene | Aromatics |
| 165 | 1.68E+02 | 1.26E+03 | 1.15E+03 | 1-Undecene, 9-methyl- | Hydrocarbons |
| 166 | 1.68E+02 | 1.01E+03 | 1.20E+03 | 2-Butenoic acid, 3-hexenyl ester, (E,Z)- | Ester |
| 167 | 1.68E+02 | 1.15E+03 | 1.17E+03 | Lilac Aldehyde D | Aldehyde |
| 168 | 1.68E+02 | 1.24E+03 | 1.29E+03 | 7-Oxabicyclo[4.1.0]heptane, 1-methyl-4-(2-methyloxiranyl)- | Terpenoids |
| 169 | 1.67E+02 | 1.12E+03 | - | diethylboryl-.delta.-Valerolactam | Heterocyclic compound |
| 170 | 1.66E+02 | 1.12E+03 | 1.23E+03 | 1-(1-Ethyl-2,3-dimethyl-cyclopent-2-enyl)-ethanone | Ketone |
| 171 | 1.66E+02 | 9.21E+02 | 1.14E+03 | 2,7-Nonadien-5-one, 4,6-dimethyl- | Ketone |
| 172 | 1.66E+02 | 1.10E+03 | 1.21E+03 | 1-(Furan-2-yl)-2-methylpentan-1-one | Ketone |
| 173 | 1.66E+02 | 1.59E+03 | 1.58E+03 | Fluorene | Aromatics |
| 174 | 1.66E+02 | 1.37E+03 | 1.37E+03 | 4-Methoxy-Benzoic acid, methyl ester | Ester |
| 175 | 1.66E+02 | 1.48E+03 | 1.65E+03 | Benzenepropanoic Acid, 4-hydroxy- | Acid |
| 176 | 1.64E+02 | 1.35E+03 | 1.45E+03 | trans-Isoeugenol | Phenol |
| 177 | 1.64E+02 | 1.45E+03 | 1.38E+03 | 3,6-Dimethyl-4H-furo[3,2-c]pyran-4-one | Heterocyclic Compound |
| 178 | 1.60E+02 | 1.37E+03 | 1.34E+03 | 2-Acetylbenzofuran | Heterocyclic compound |
| 179 | 1.58E+02 | 1.27E+03 | 1.28E+03 | Nonanoic Acid | Acid |
| 180 | 1.58E+02 | 1.12E+03 | 1.04E+03 | Octanoic acid, methyl ester | Ester |
| 181 | 1.56E+02 | 1.21E+03 | 1.21E+03 | Decanal | Aldehyde |
| 182 | 1.56E+02 | 1.44E+03 | 1.45E+03 | Naphthalene, 1,2-dimethyl- | Aromatics |
| 183 | 1.55E+02 | 1.26E+03 | 1.26E+03 | 3,4-Methylpropylsuccinimide | Heterocyclic compound |
| 184 | 1.54E+02 | 1.10E+03 | 1.10E+03 | Linalool | Terpenoids |
| 185 | 1.54E+02 | 1.01E+03 | 1.14E+03 | Menthen-4-ol | Alcohol |
| 186 | 1.54E+02 | 1.25E+03 | 1.26E+03 | 2,6-Octadien-1-ol, 3,7-dimethyl- | Alcohol |
| 187 | 1.54E+02 | 1.18E+03 | 1.18E+03 | 3-Cyclohexen-1-ol, 4-methyl-1-(1-methylethyl)-, (R)- | Terpenoids |
| 188 | 1.54E+02 | 1.14E+03 | 1.18E+03 | Terpinen-4-ol | Terpenoids |
| 189 | 1.54E+02 | 1.20E+03 | 1.19E+03 | L-alpha-Terpineol | Terpenoids |
| 190 | 1.54E+02 | 1.25E+03 | 1.09E+03 | 3-Methyl-2-butenoic acid, cyclobutyl ester | Ester |
| 191 | 1.54E+02 | 1.38E+03 | 1.49E+03 | 1,8-Ethylenenaphthalene | Aromatics |
| 192 | 1.52E+02 | 1.31E+03 | 1.19E+03 | 3,5-Dimethyl-1-butylpyrazole | Heterocyclic compound |
| 193 | 1.52E+02 | 1.19E+03 | 1.24E+03 | Dill ether | Heterocyclic compound |
| 194 | 1.52E+02 | 1.17E+03 | 1.33E+03 | 1-Adamantanol | Alcohol |
| 195 | 1.52E+02 | 1.22E+03 | 1.23E+03 | 3-Cyclohexene-1-acetAldehyde, .alpha.,4-dimethyl- | Aldehyde |
| 196 | 1.52E+02 | 1.27E+03 | 1.14E+03 | 3,7-Nonadien-2-one, 8-methyl-, (E)- | Ketone |
| 197 | 1.52E+02 | 1.19E+03 | 1.13E+03 | 1-(1,2,3-Trimethyl-cyclopent-2-enyl)-ethanone | Ketone |
| 198 | 1.52E+02 | 1.32E+03 | 1.12E+03 | L-Fenchone | Terpenoids |
| 199 | 1.52E+02 | 1.14E+03 | 1.14E+03 | 2,6,6-Trimethyl-2-cyclohexene-1,4-dione | Ketone |
| 200 | 1.52E+02 | 1.40E+03 | 1.54E+03 | 2,4-dihydroxy-6-methyl-BenzAldehyde | Aldehyde |
| 201 | 1.52E+02 | 1.72E+03 | 1.45E+03 | Benzoic Acid, 4-methoxy- | Aromatics |
| 202 | 1.51E+02 | 1.34E+03 | 1.34E+03 | Methyl anthranilate | Ester |
| 203 | 1.51E+02 | 1.58E+03 | 1.46E+03 | 1,4-enzodioxan-6-amine | Heterocyclic compound |
| 204 | 1.51E+02 | 1.59E+03 | 1.48E+03 | Pyrolo[3,2-d]pyrimidin-2,4(1H,3H)-dione | Ketone |
| 205 | 1.50E+02 | 1.62E+03 | 1.53E+03 | N,4,5-trimethyl-Phenyl-1,2-diAmine | Amine |
| 206 | 1.50E+02 | 1.15E+03 | 1.16E+03 | Pyrazine, 3,5-diethyl-2-methyl- | Heterocyclic compound |
| 207 | 1.50E+02 | 1.20E+03 | 1.30E+03 | 1,3-Cyclohexadiene-1-carboxaldehyde, 2,6,6-trimethyl- | Terpenoids |
| 208 | 1.50E+02 | 1.16E+03 | 1.16E+03 | Acetic acid, phenylmethyl ester | Ester |
| 209 | 1.50E+02 | 1.31E+03 | 1.36E+03 | Ethanone, 1-(2-hydroxy-5-methylphenyl)- | Ketone |
| 210 | 1.50E+02 | 1.32E+03 | 1.31E+03 | BenzAldehyde, 4-ethoxy- | Aldehyde |
| 211 | 1.48E+02 | 1.47E+03 | 1.26E+03 | 3-Methylbenzothiophene | Heterocyclic compound |
| 212 | 1.44E+02 | 1.16E+03 | 1.15E+03 | 1-Nonanol | Alcohol |
| 213 | 1.44E+02 | 1.18E+03 | 1.28E+03 | Octanoic Acid | Acid |
| 214 | 1.44E+02 | 1.17E+03 | 1.23E+03 | 3-phenyl-Furan | Heterocyclic compound |
| 215 | 1.42E+02 | 1.05E+03 | 9.31E+02 | 3-Ethyl-3-methylheptane | Hydrocarbons |
| 216 | 1.42E+02 | 1.10E+03 | 1.10E+03 | Nonanal | Aldehyde |
| 217 | 1.42E+02 | 1.24E+03 | 1.24E+03 | n-Caproic acid vinyl ester | Ester |
| 218 | 1.42E+02 | 9.90E+02 | 9.74E+02 | 4-Penten-1-ol, propanoate | Ester |
| 219 | 1.42E+02 | 1.30E+03 | 1.30E+03 | Naphthalene, 2-methyl- | Aromatics |
| 220 | 1.40E+02 | 1.04E+03 | 1.09E+03 | Cyclohexanone, 2,2,6-trimethyl- | Ketone |
| 221 | 1.40E+02 | 1.20E+03 | 1.26E+03 | 2,5-ThiophenedicarboxAldehyde | Aldehyde |
| 222 | 1.39E+02 | 1.23E+03 | 1.24E+03 | 1H-Pyrrole-2,5-dione, 3-ethyl-4-methyl- | Ketone |
| 223 | 1.38E+02 | 1.05E+03 | 9.91E+02 | 2,6-Dimethyl-2-trans-6-octadiene | Terpenoids |
| 224 | 1.38E+02 | 1.21E+03 | 1.01E+03 | 2-Dimethylamino-4-methyl-pent-4-enenitrile | Others |
| 225 | 1.38E+02 | 9.90E+02 | 9.93E+02 | Furan, 2-pentyl- | Heterocyclic compound |
| 226 | 1.38E+02 | 1.15E+03 | 1.15E+03 | 2,6-Nonadienal, (E,Z)- | Aldehyde |
| 227 | 1.37E+02 | 1.26E+03 | 1.26E+03 | 1H-Pyrrole-2,5-dione, 3-ethenyl-4-methyl- | Ketone |
| 228 | 1.36E+02 | 1.05E+03 | 1.05E+03 | .beta.-Phellandrene | Terpenoids |
| 229 | 1.36E+02 | 1.24E+03 | 1.05E+03 | trans-.beta.-Ocimene | Terpenoids |
| 230 | 1.36E+02 | 1.28E+03 | 1.08E+03 | Cyclohexene, 4-methyl-1-(1-methylethenyl)- | Hydrocarbons |
| 231 | 1.36E+02 | 1.08E+03 | 1.08E+03 | Pyrazine, 3-ethyl-2,5-dimethyl- | Heterocyclic compound |
| 232 | 1.36E+02 | 1.23E+03 | 1.08E+03 | 2,6-diethyl-Pyrazine | Heterocyclic compound |
| 233 | 1.36E+02 | 1.10E+03 | 1.10E+03 | Benzoic acid, methyl ester | Ester |
| 234 | 1.36E+02 | 1.23E+03 | 1.46E+03 | Acetophenone, 4'-hydroxy- | Ketone |
| 235 | 1.36E+02 | 1.25E+03 | 1.26E+03 | Benzeneacetic acid | Acid |
| 236 | 1.35E+02 | 1.23E+03 | 1.24E+03 | Benzothiazole | Heterocyclic compound |
| 237 | 1.34E+02 | 1.03E+03 | 1.04E+03 | Benzene, 1-methyl-3-(1-methylethyl)- | Aromatics |
| 238 | 1.34E+02 | 1.13E+03 | 1.03E+03 | p-Cymene | Aromatics |
| 239 | 1.34E+02 | 1.15E+03 | 1.21E+03 | BenzAldehyde, 2,4-dimethyl- | Aldehyde |
| 240 | 1.34E+02 | 1.23E+03 | - | 2-Methylthiolane, S,S-dioxide | Sulfur compounds |
| 241 | 1.31E+02 | 1.41E+03 | 1.39E+03 | Indole, 3-methyl- | Heterocyclic compound |
| 242 | 1.30E+02 | 1.06E+03 | 9.95E+02 | 2-Ethyl-1-hexanol | Alcohol |
| 243 | 1.30E+02 | 1.08E+03 | 1.08E+03 | Heptanoic Acid | Acid |
| 244 | 1.30E+02 | 9.23E+02 | 1.00E+03 | Hexanoic acid, methyl ester | Ester |
| 245 | 1.28E+02 | 1.11E+03 | 1.11E+03 | Cyclohexanol, 2,6-dimethyl- | Alcohol |
| 246 | 1.27E+02 | 1.11E+03 | 1.20E+03 | 2-Thienylamide | Heterocyclic compound |
| 247 | 1.26E+02 | 9.74E+02 | 9.85E+02 | 3,5,5-Trimethyl-2-Hexene | Hydrocarbons |
| 248 | 1.26E+02 | 9.83E+02 | 9.86E+02 | 5-Hepten-2-one, 6-methyl- | Ketone |
| 249 | 1.26E+02 | 1.01E+03 | 1.02E+03 | Benzyl chloride | Halogenated hydrocarbons |
| 250 | 1.26E+02 | 9.69E+02 | 9.74E+02 | Dimethyl triSulfur compounds | Sulfur compounds |
| 251 | 1.24E+02 | 1.07E+03 | 1.10E+03 | 3,5-Octadien-2-one | Ketone |
| 252 | 1.24E+02 | 8.83E+02 | 8.93E+02 | 2-n-Butyl furan | Heterocyclic compound |
| 253 | 1.24E+02 | 1.10E+03 | 1.04E+03 | 2-Acetyl-5-methylfuran | Heterocyclic compound |
| 254 | 1.23E+02 | 1.18E+03 | 1.17E+03 | 3-Formyl-4,5-dimethyl-pyrrole | Heterocyclic compound |
| 255 | 1.22E+02 | 8.41E+02 | 8.56E+02 | 1,3-Cyclopentadiene, 5,5-dimethyl-1-ethyl- | Hydrocarbons |
| 256 | 1.22E+02 | 1.00E+03 | 9.93E+02 | Pyrazine, 2-ethyl-5-methyl- | Heterocyclic compound |
| 257 | 1.22E+02 | 1.18E+03 | 1.28E+03 | Benzoic acid | Aromatics |
| 258 | 1.20E+02 | 1.07E+03 | 9.70E+02 | Benzene, 1-ethyl-2-methyl- | Aromatics |
| 259 | 1.20E+02 | 1.22E+03 | 1.22E+03 | 2,3-dihydro-Benzofuran | Heterocyclic compound |
| 260 | 1.20E+02 | 1.04E+03 | 1.05E+03 | BenzeneacetAldehyde | Aldehyde |
| 261 | 1.20E+02 | 1.02E+03 | 9.20E+02 | DiSulfur compounds, methyl 2-propenyl | Sulfur compounds |
| 262 | 1.18E+02 | 9.02E+02 | 9.06E+02 | Ethanol, 2-butoxy- | Alcohol |
| 263 | 1.17E+02 | 1.29E+03 | 1.20E+03 | m-Aminophenylacetylene | Amine |
| 264 | 1.16E+02 | 1.05E+03 | 1.13E+03 | (S)-(+)-2',3'-Dideoxyribonolactone | Heterocyclic compound |
| 265 | 1.14E+02 | 1.00E+03 | 9.82E+02 | 4-Hexenoic Acid | Acid |
| 266 | 1.14E+02 | 1.04E+03 | 1.05E+03 | 2,4-Imidazolidinedione, 1-methyl- | Heterocyclic compound |
| 267 | 1.13E+02 | 2.38E+03 | - | Ethyl isocyanoacetate | Ester |
| 268 | 1.12E+02 | 9.61E+02 | 1.02E+03 | Dehydromevalonic lactone | Aromatics |
| 269 | 1.10E+02 | 9.95E+02 | 8.41E+02 | Furan, 2-propyl- | Heterocyclic compound |
| 270 | 1.10E+02 | 1.06E+03 | 9.62E+02 | 2-Cyclohexen-1-one, 3-methyl- | Ketone |
| 271 | 1.10E+02 | 1.03E+03 | 1.28E+03 | Hydroquinone | Aromatics |
| 272 | 1.09E+02 | 1.06E+03 | 1.04E+03 | 3-Acetyl-1H-pyrroline | Heterocyclic compound |
| 273 | 1.08E+02 | 9.10E+02 | 9.17E+02 | Pyrazine, 2,5-dimethyl- | Heterocyclic compound |
| 274 | 1.08E+02 | 1.03E+03 | 1.04E+03 | Benzyl Alcohol | Alcohol |
| 275 | 1.06E+02 | 8.90E+02 | 8.65E+02 | p-Xylene | Aromatics |
| 276 | 1.06E+02 | 9.62E+02 | 9.60E+02 | BenzAldehyde | Aldehyde |
| 277 | 1.04E+02 | 8.90E+02 | 8.93E+02 | Styrene | Aromatics |
| 278 | 1.03E+02 | 9.80E+02 | 8.54E+02 | 3-methyl-Thiazolidine | Heterocyclic compound |
| 279 | 1.02E+02 | 8.58E+02 | 8.73E+02 | Butanoic Acid, 2-methyl- | Acid |
| 280 | 1.02E+02 | 8.49E+02 | 8.77E+02 | Butanoic Acid, 3-methyl- | Acid |
| 281 | 1.02E+02 | 8.86E+02 | 9.11E+02 | Pentanoic Acid | Acid |
| 282 | 1.00E+02 | 7.95E+02 | 8.01E+02 | Hexanal | Aldehyde |
| 283 | 9.81E+01 | 8.51E+02 | 8.56E+02 | 2-Hexenal | Aldehyde |
| 284 | 9.80E+01 | 8.51E+02 | 8.30E+02 | Maleic anhydride | Heterocyclic compound |
| 285 | 9.60E+01 | 8.29E+02 | 8.32E+02 | 3-FurAldehyde | Aldehyde |
| 286 | 9.40E+01 | 9.11E+02 | 9.80E+02 | Phenol | Phenol |
| 287 | 9.21E+01 | 7.58E+02 | 7.73E+02 | Toluene | Aromatics |
| 288 | 8.81E+01 | 7.78E+02 | 7.18E+02 | Acetoin | Aldehyde |
| 289 | 8.61E+01 | 7.63E+02 | 7.67E+02 | 2-Penten-1-ol, (Z)- | Alcohol |
| 290 | 8.61E+01 | 7.66E+02 | 7.05E+02 | tetrahydro-3-methyl-Furan | Heterocyclic compound |
| 291 | 8.50E+01 | 1.17E+03 | - | Phosphoramidous difluoride | Halogenated hydrocarbons |
| 292 | 8.41E+01 | 7.46E+02 | 7.61E+02 | Furan, 2,3-dihydro-4-methyl- | Heterocyclic compound |
| 293 | 8.40E+01 | 8.27E+02 | 9.14E+02 | 2(3H)-Furanone | Ketone |
| 294 | 8.20E+01 | 8.54E+02 | - | Methanesulfinyl fluoride | Halogenated hydrocarbons |
| 295 | 7.40E+01 | 9.01E+02 | 7.55E+02 | Glycidol | Alcohol |
